# Topologically Structured PLLA Fibers With Stress Concentration Effects for Health Monitoring

**DOI:** 10.1002/advs.75619

**Published:** 2026-05-11

**Authors:** Longfei Li, Juwei Yang, Yiqian Wang, Qiao Yu, Zhenmin Fan, Chang Zhu, Ming Yin, Wei Hua, Zhou Li

**Affiliations:** ^1^ Beijing Institute of Nanoenergy and Nanosystems Chinese Academy of Sciences Beijing China; ^2^ School of Nanoscience and Engineering University of Chinese Academy of Sciences Beijing China; ^3^ Vita Tech Innovation Center School of Clinical Medicine Tsinghua Changgung Hospital Tsinghua University Beijing China; ^4^ National Center for Cardiovascular Diseases Fuwai Hospital Chinese Academy of Medical Sciences and Peking Union Medical College Beijing China; ^5^ School of Biomedical Engineering Tsinghua University Beijing China

**Keywords:** biomedical engineering, flexible electronic devices, piezoelectric enhancement strategy, poly(L‐lactic acid) fiber, stress concentration

## Abstract

The development of flexible electronic devices necessitates materials with high piezoelectric performance. Poly(L‐lactic acid) (PLLA) nanofiber‐based piezoelectric membranes hold promise for self‐powered health monitoring and tissue repair, yet their intrinsic piezoelectric performance remains insufficient for practical applications. Here, we develop a multi‐path strategy to enhance the piezoelectric properties through crystallography and hierarchical design of PLLA fibers. We also demonstrate that incorporating high‐aspect‐ratio needle‐like hydroxyapatite (HAp) into PLLA optimizes crystallinity and enhances piezoelectric output. Building upon this, we construct PLLA‐HAp/PLLA composite fiber membranes with topological structures to further amplify the piezoelectric effect. Notably, low‐concentration HAp dispersed within random PLLA fibers induces stress concentration at the interface with aligned PLLA fibers, resulting in satisfactory piezoelectric performance. In the impact mode and bending mode, the piezoelectric output is approximately 6 and 14 times that of pure PLLA fiber, respectively. The optimized fiber membranes demonstrated excellent potential for monitoring human physiological activities, as validated by subsequent experiments that successfully recorded porcine joint movements and heart rates. This piezoelectric enhancement strategy offers a new approach for next‐generation high‐performance piezoelectric devices, with broad applicability in health monitoring and tissue engineering.

## Graphical abstract

In this study, a multi‐path strategy was developed to enhance the piezoelectric properties of PLLA fibers through the stress concentration effect. The optimized devices generate piezoelectric output up to 14 times that of pure PLLA fibers. This piezoelectric enhancement strategy offers a new approach for high‐performance piezoelectric devices, with broad applicability in health monitoring and tissue engineering.

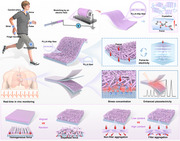

## 1 Introduction

Flexible electronic devices hold revolutionary potential in health monitoring and medical diagnostics [1]. Their mechanical compatibility with human tissue and ability to function without external power sources enable real‐time, non‐invasive physiological signal monitoring [2]. This personalized health management approach not only provides early warnings of disease risks but also offers crucial support for rehabilitation therapy and precision medicine [3]. For instance, flexible sensors can monitor heart rate, blood pressure, and muscle activity, aiding early diagnosis of cardiovascular diseases or chronic conditions [4, 5]. The generation of these electrical signals is closely tied to the piezoelectric properties of flexible devices‐their ability to convert mechanical energy into electrical energy [6]. The development and performance optimization of piezoelectric materials are key to achieving breakthroughs in flexible healthcare monitoring devices [7].

Currently, piezoelectric materials can be generally categorized into two main types: inorganic piezoelectric ceramics and organic piezoelectric polymers [8]. Traditional piezoelectric ceramics, despite their outstanding piezoelectric properties, suffer from inherent drawbacks such as brittleness and potential biotoxicity, thereby limiting their application in biomedical fields [9]. In contrast, organic piezoelectric polymers, particularly poly(L‐lactic acid) (PLLA), demonstrate significant potential for health monitoring and tissue repair due to their excellent biocompatibility, biodegradability, and superior flexibility [10, 11]. The piezoelectricity of PLLA primarily originates from the arrangement of dipoles along its molecular chains [12]. Its relatively low piezoelectricity derives from the fact that dipoles on C═O groups are bound by covalent bonds rather than the ionic bonds commonly found in inorganic piezoelectric materials [13]. Therefore, PLLA itself exhibits poor charge separation capability. Additionally, PLLA is a semi‐crystalline polymer, and its piezoelectricity primarily originates from charge separation induced by deformation in its crystalline regions [14]. However, the oriented dipoles within the amorphous regions can also generate piezoelectric effects [15]. Notably, stress transmission is hindered in the amorphous regions, resulting in significant mechanical losses that further compromise piezoelectric performance [13]. Consequently, enhancing PLLA's piezoelectric properties poses an urgent challenge.

To date, various strategies have been developed to enhance the piezoelectric properties of PLLA. For instance, piezoelectric coupling mechanisms utilize high‐piezoelectric‐coefficient materials, such as barium titanate, as fillers to amplify PLLA's piezoelectric response [16]. It is also possible to employ interfacial interaction strategies, such as utilizing the intermolecular interactions between the ─OH group of glycine and the C═O group on PLLA [17], to enhance the piezoelectric properties of PLLA. The electrical output is positively correlated with the perceived force. Amplifying the perceived force at the mechanical structural level to improve the piezoelectric effect is another promising approach [18]. Given that the piezoelectric properties of PLLA are closely related to its crystallinity and molecular chain orientation during processing, research exploring the role of additives as heterogeneous nucleation centers to promote PLLA crystallization holds promising prospects [19]. For example, zoledronic acid effectively enhanced the piezoelectric coefficient of PLLA by acting as a nucleating agent [20]. Indeed, previous studies have demonstrated that morphologically anisotropic hydroxyapatite (HAp) particles, particularly those with a high aspect ratio, can significantly influence PLLA crystallization and polymer chain orientation, thereby enhancing its piezoelectric properties by acting as nucleating agents [21]. Building upon these fundamental insights, our current work extends this understanding by exploring how specific topological structures and engineered stress concentration effects, combined with HAp‐mediated crystallization, can further amplify the piezoelectric response of PLLA fibers. To achieve oriented alignment of PLLA molecular chains, processing methods including 3D printing [22], thermal stretching [23], melt spinning [24], and electrospinning [25, 26] are being explored. Among these, the high electric field generated by electrospinning technology drives the dipole groups to align perpendicular to the PLLA fiber length [27]. By optimizing the electrospinning process parameters, the fiber diameter, morphology, and degree of molecular chain orientation can be effectively controlled [28], enabling the preparation of PLLA nanofiber membranes with ideal piezoelectric properties.

Inspired by the hierarchical architecture and inherent piezoelectricity of bone tissue, which forms through the self‐assembly of collagen and HAp [29], we hypothesize that leveraging specific structural designs could enhance PLLA's piezoelectric performance. The bioelectrical properties of bone are primarily attributed to collagen, and the piezoelectricity arising from non‐centrosymmetric conformations caused by the relative sliding of collagen fibers [30], but HAp also plays a regulatory role in these properties. Some research has explored the electrical properties of HAp [31], suggesting potential piezoelectric responses associated with ion displacements and changes in dipole moments within its crystal structure under specific conditions [32, 33]. However, HAp is still predominantly considered a non‐piezoelectric component in mainstream research. Nevertheless, it is undeniable that HAp crystals influence the transmission of mechanical loads on bone, thereby affecting the mechanical response of collagen fibers to tensile or compressive forces. This factor ultimately determines the generation of piezoelectric effects [34]. Here, we hypothesize that the properties of HAp can be leveraged to achieve multiple enhancement pathways for PLLA fibers: On one hand, HAp fillers serve as heterogeneous nucleation sites, improving PLLA's crystallization and thereby enhancing the piezoelectric response. On the other hand, the topological structure of the PLLA membrane is designed to amplify the perception force through stress transfer and concentration effects at the fiber interface, thereby further enhancing the piezoelectric effect.

**FIGURE 1:**
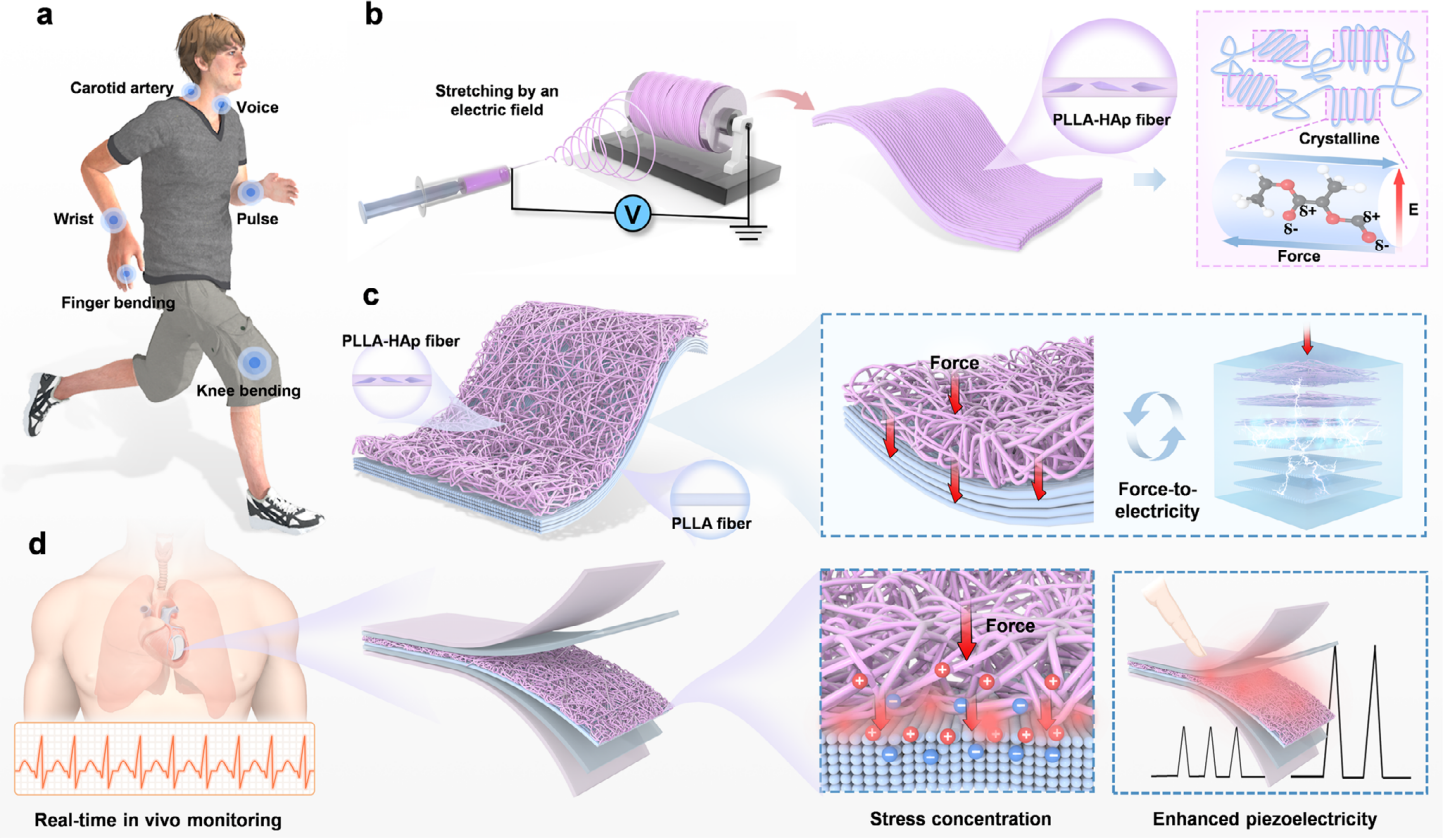
Schematic of an enhanced piezoelectric membrane based on PLLA fibers for health monitoring. (a) Monitoring of human physiological signals. (b) HAp enhances the piezoelectric properties of electrospun PLLA fibers by increasing their crystallinity. (c) Schematic of force‐to‐electricity conversion in PLLA‐HAp/PLLA topological fiber membranes with stress concentration effects. (d) The fiber membrane with piezoelectric enhancement effect for real‐time monitoring of cardiac activity.

Based on these insights, we have developed multiple piezoelectric enhancement strategies for PLLA nanofiber devices in physiological monitoring (Figure 1). By examining HAp with different morphologies, it was determined that doping needle‐like HAp with a high aspect ratio endows PLLA fibers with optimal crystallinity and piezoelectricity. Subsequent optimization of piezoelectric performance was achieved by constructing PLLA‐HAp/PLLA composite membranes with diverse topological structures. Low‐concentration HAp dispersed within random PLLA fibers induces stress concentration effects at the interface with aligned PLLA fibers, resulting in satisfactory piezoelectric performance. The optimized fiber membrane enables monitoring of human physiological activities and recording of joint movements and heart rate in pigs. This presented piezoelectric enhancement strategy offers new insights for next‐generation high‐performance piezoelectric devices, with potential applications in health monitoring and tissue repair.

## 2 Results and Discussions

### 2.1 Fabrication and Characterization of the PLLA‐HAp Fiber Membrane

**FIGURE 2:**
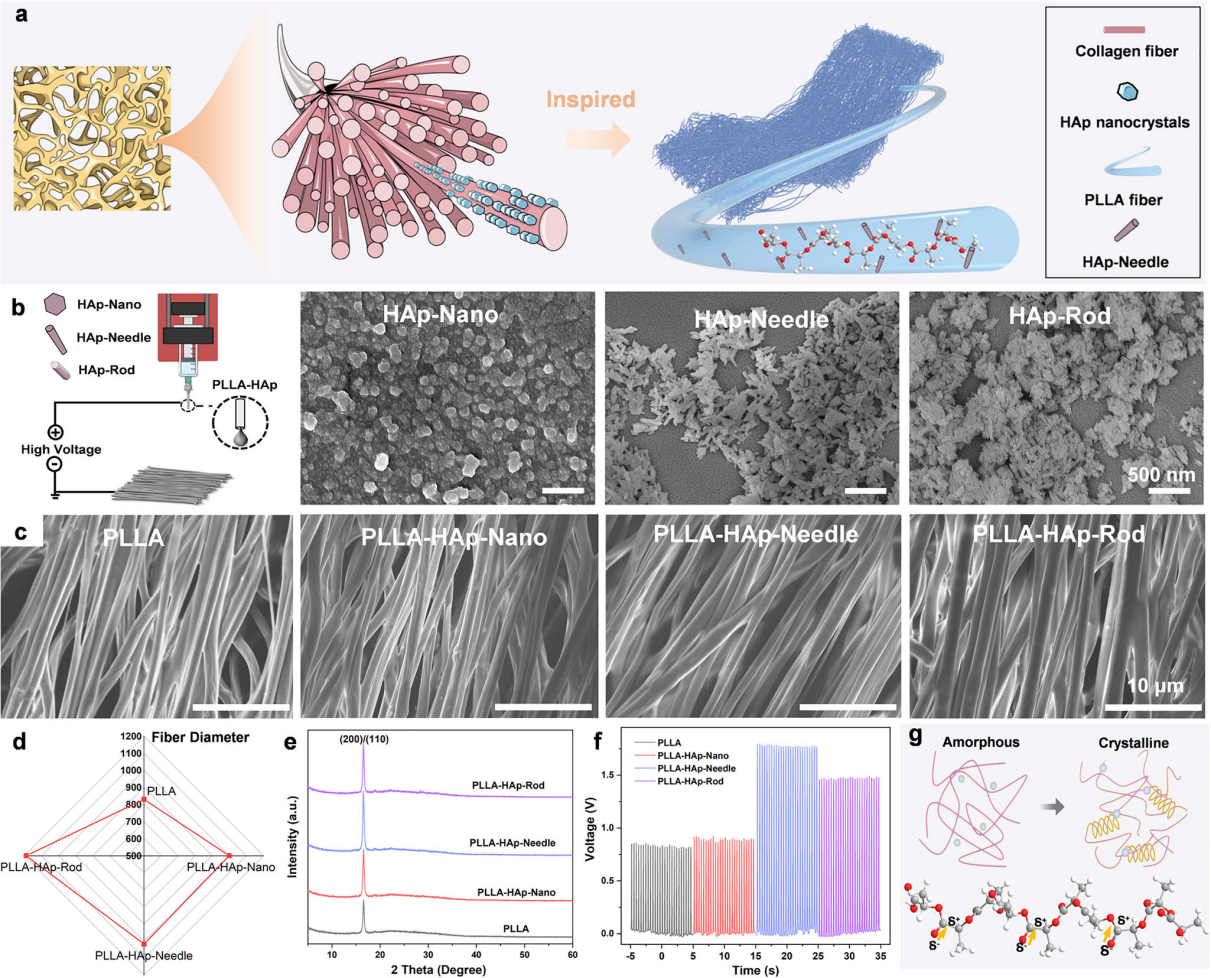
PLLA‐HAp fiber membranes doped with HAp of different morphologies. (a) Illustration of PLLA‐HAp fiber design inspired by the piezoelectricity of collagen fibers and HAp structure. SEM images of (b) HAp with different morphologies and (c) PLLA‐HAp fibers after doping. (d) Average diameter and (e) XRD curve of various PLLA‐HAp fibers and PLLA fibers. (f) Piezoelectric output of each group of fiber membranes. (g) A schematic diagram illustrating HAP's enhancement of PLLA crystallinity and the orientation of dipole moments along PLLA molecular chains.

As mentioned above, HAP and collagen fibers play a crucial role in bone tissue [35]. PLLA fibers and collagen fibers exhibit similar piezoelectric properties. Based on this, we first investigated HAp's ability to modulate the piezoelectricity of PLLA fibers (Figure 2a). We selected three distinct morphologies of HAp (nanoparticles, needles, and rods) and incorporated them into PLLA electrospun fibers to gain deeper insights into how HAp morphology modulates the microstructure and piezoelectric properties of PLLA fibers (Figure 2b). Specifically, incorporating HAp with different morphologies into electrospun PLLA fibers revealed that HAp doping did not affect the surface morphology of PLLA fibers but altered their diameter distribution (Figure 2c). PLLA fibers doped with needle‐ and rod‐shaped HAp exhibited average diameters ranging from 1000 to 1200 nm (Figure 2d; Figure S1). This phenomenon may be attributed to changes in solution rheological properties during electrospinning, likely caused by the high aspect ratio of the HAp particles.

Interestingly, XRD results indicate that compared to pure PLLA, PLLA‐HAp‐Needle and PLLA‐HAp‐Rod groups exhibit a higher crystalline peak at 16.5°, confirming the presence of the (200) and (110) crystal planes corresponding to the *β*‐phase structure (Figure 2e) [36]. The fact that the HAp diffraction peaks are barely visible may be due to the relatively low doping concentration of HAp in each fiber group, resulting in weak diffraction signals. This is because the PLLA matrix itself exhibits very strong diffraction peaks (near 16.5°), which may significantly mask or completely obscure the weaker HAp diffraction peaks at low concentrations. We also observed that when Needle‐HAp was dispersed in hexafluoroisopropanol (HFP), the intensity of its crystalline peaks was reduced compared to that of the Needle‐HAp powder (as shown in Figure S2). This suggests that the solvent may affect the crystallinity or dispersion state of HAp, thereby further weakening its diffraction intensity. However, in PLLA‐HAp‐Needle fibers, faint diffraction peaks can be observed around 26° and 32°, which correspond to the (002) and (211) planes of HAp, respectively. The increase in PLLA crystallinity induced by HAp had a beneficial effect on the piezoelectric properties, and the piezoelectric outputs from each group also confirmed this hypothesis. As shown in Figure 2f, compared with pure PLLA (0.85 V), the output of PLLA‐HAp‐Nano fibers showed almost no improvement. However, both PLLA‐HAp‐Needle and PLLA‐HAp‐Rod fibers exhibited significant increases in piezoelectric output. Notably, PLLA‐HAp‐Needle fibers achieved an output of 1.75 V. Similar results were also obtained when evaluating the charges and surface charge densities of each group (Figure S3). This may be attributed to the needle‐like and rod‐like HAp with high aspect ratios acting as nucleating agents, providing more nucleation sites during PLLA crystallization. This promotes the ordered arrangement of PLLA molecular chains, thereby forming more *β*‐phase crystalline structures (Figure 2g). To exclude the electrical effects of HAp, the voltage output of non‐piezoelectric polylactic acid (PLA) fiber membranes, as well as PLA‐HAp and PLLA‐HAp fiber membranes, was measured under identical mechanical stimulation conditions. Figure S4 shows that there is no significant difference in piezoelectric output between the presence and absence of HAp on a non‐piezoelectric polymer substrate; both were far lower than the output capacity of PLLA‐HAp. These strongly demonstrate that HAp itself does not possess significant direct piezoelectricity or charge generation capability, but is used as a filler without piezoelectric properties. Our further studies on concentration dependence also indicate that the contribution of these heterogeneous nucleation sites is closely related to the HAp doping concentration. As shown in Figure S5, doping with 1 wt.% HAp slightly promotes the crystallization of PLLA, reaching a peak at 3%, then decreasing at 5 wt.%. Consistent with previous findings, low‐concentration doping of HAp with a high aspect ratio is beneficial for PLLA crystallization and piezoelectric response [21]. The doping concentration may be related to the preparation process. This extended study on the effect of HAp concentration on electrospun PLLA fibers offers significant insights into the structure‐property relationship, informing the optimal design of PLLA and other composite systems for high‐performance piezoelectric devices.

### 2.2 Fabrication and Characterization of the Composite Fiber Membrane

**FIGURE 3:**
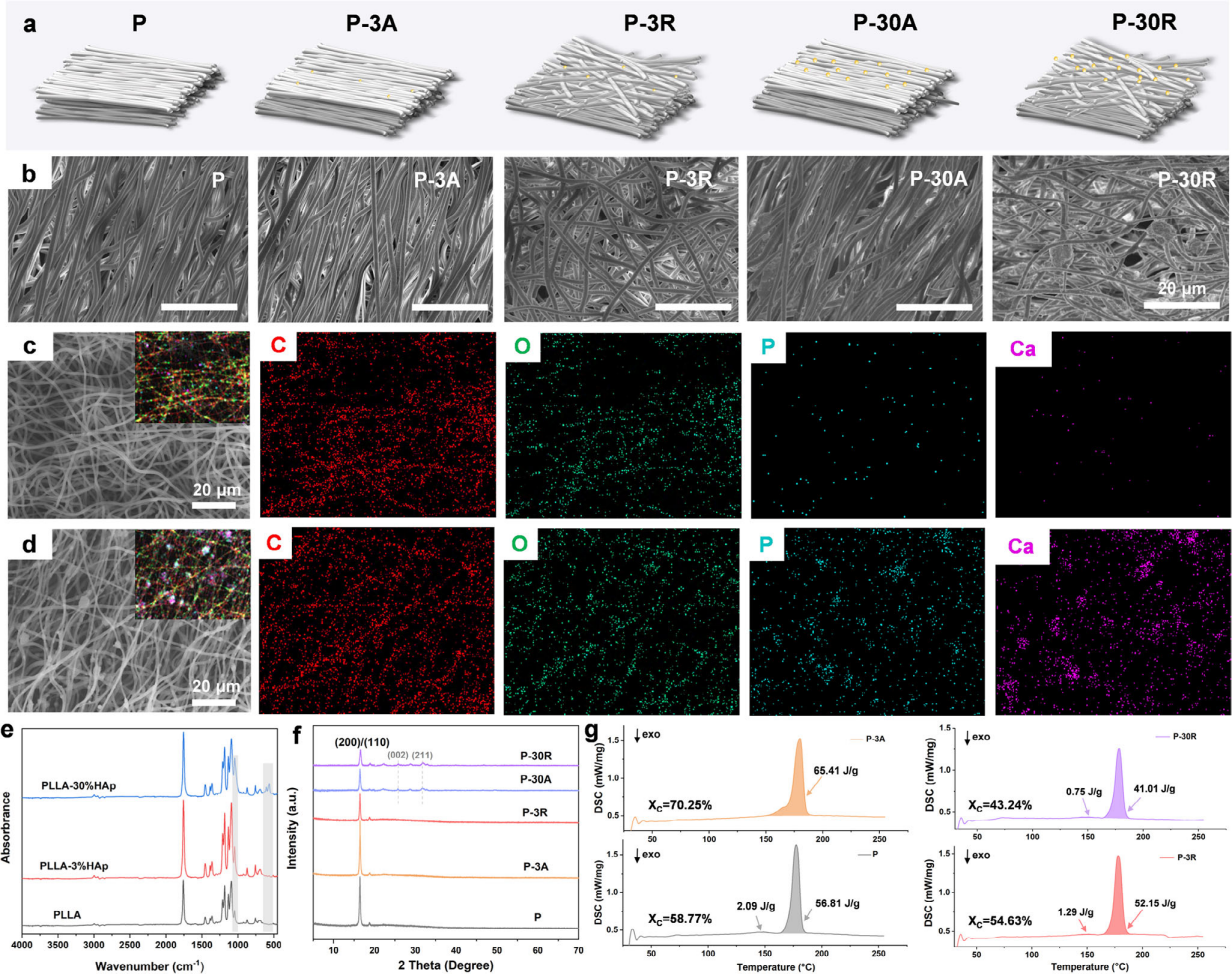
Constructing fiber membranes with different topological structures. (a) Illustration of PLLA‐HAp fiber design inspired by the piezoelectricity of bone tissue. (b) SEM images of fiber membranes with different topologies. Elements mapping for (c) P‐3R and (d) P‐30R fiber membranes. (e) FTIR images of PLLA, PLLA‐HAP (3 wt.%), and PLLA‐HAP (30 wt.%) fiber membranes. (f) XRD curve of different fiber membranes. (g) Piezoelectric output of each group of fiber membranes. (g) DSC curves and crystallinity (X_C_) for P, P‐3A, P‐3R, and P‐30R fiber membranes.

Considering the hierarchical structure of bone and the potential mechanisms by which HAp transmits mechanical forces within it, we constructed several topological structures to verify the effects generated by HAp within PLLA fibers (Figure 3a). The following topologies were built upon the aligned PLLA fibers: aligned PLLA fibers (P), low‐concentration‐doped aligned PLLA‐HAp fibers (P‐3A), low‐concentration‐doped random PLLA‐HAp fibers (P‐3R), high‐concentration‐doped aligned PLLA‐HAp fibers (P‐30A), and high‐concentration‐doped random PLLA‐HAp fibers (P‐30R). Among these, the P, P‐3A, and P‐30A groups exhibited ordered fiber morphology, while the P‐3R and P‐30R groups displayed random fiber structures (Figure 3b). High‐concentration doping results in HAp agglomeration on the fiber surface, whereas no significant agglomeration was observed in the low‐concentration doping groups. The element mapping images further confirm this finding. As shown in Figure 3c,d, Ca and P elements exhibit dense distribution in P‐30R fibers, whereas they are uniformly sparse in P‐3R fibers. Figure S6 also shows the elemental distribution of P, P‐3A, and P‐30A, with elements uniformly distributed across the fibers. The new absorption peaks observed in the FTIR are located in the 900–1200 and 560–640 cm^−1^ regions, corresponding to the vibrations of phosphate and hydroxyl groups, respectively (Figure 3e). The intensity of the new peak is positively correlated with the HAp content.

These fiber membranes with varying doping concentrations and topological structures also contribute differently to the crystallization ability of PLLA. As shown in Figure 3f, the P‐3A group with an aligned fiber structure and low‐concentration doping exhibits enhanced PLLA crystallization. This is because high rotational speeds can stretch the initial PLLA fibers, while 3 wt.% HAp serves as nucleation sites, further improving the PLLA's crystallinity. Interestingly, P‐3R did not exhibit satisfactory crystallization performance. This may be because the HAp doped into PLLA struggles to promote PLLA crystallization without undergoing stretching. We examined the crystallization behavior of PLLA in each group using DSC. As shown in Figure 3g, compared to the crystallization behavior of pure PLLA, the P‐3A fiber membrane exhibited no significant cold crystallization during heating, only showing a melting endothermic peak (65.41 J/g). This indicates that the P‐3A fiber membrane exhibits improved crystallinity compared to other groups, and the crystallinity reached 70.25%. The crystallinity of the P‐3R group is 54.63%; this slight decrease may be related to the disordered PLLA fibers. We also studied the DSC curves of P‐3R fiber membranes at different heating rates and conducted a preliminary analysis of the crystallization kinetics (Figure S7). The DSC curves clearly show that, as the heating rate increases, the crystallization peaks shift to higher temperatures. At a 30 wt.% HAp content, it even exhibits an inhibitory effect on PLLA crystallization. This highlights that both the aligned fibers and the appropriate doping concentration are crucial factors in regulating the crystallization behavior of PLLA.

### 2.3 Piezoelectric Properties of the Composite Fiber Membrane

**FIGURE 4:**
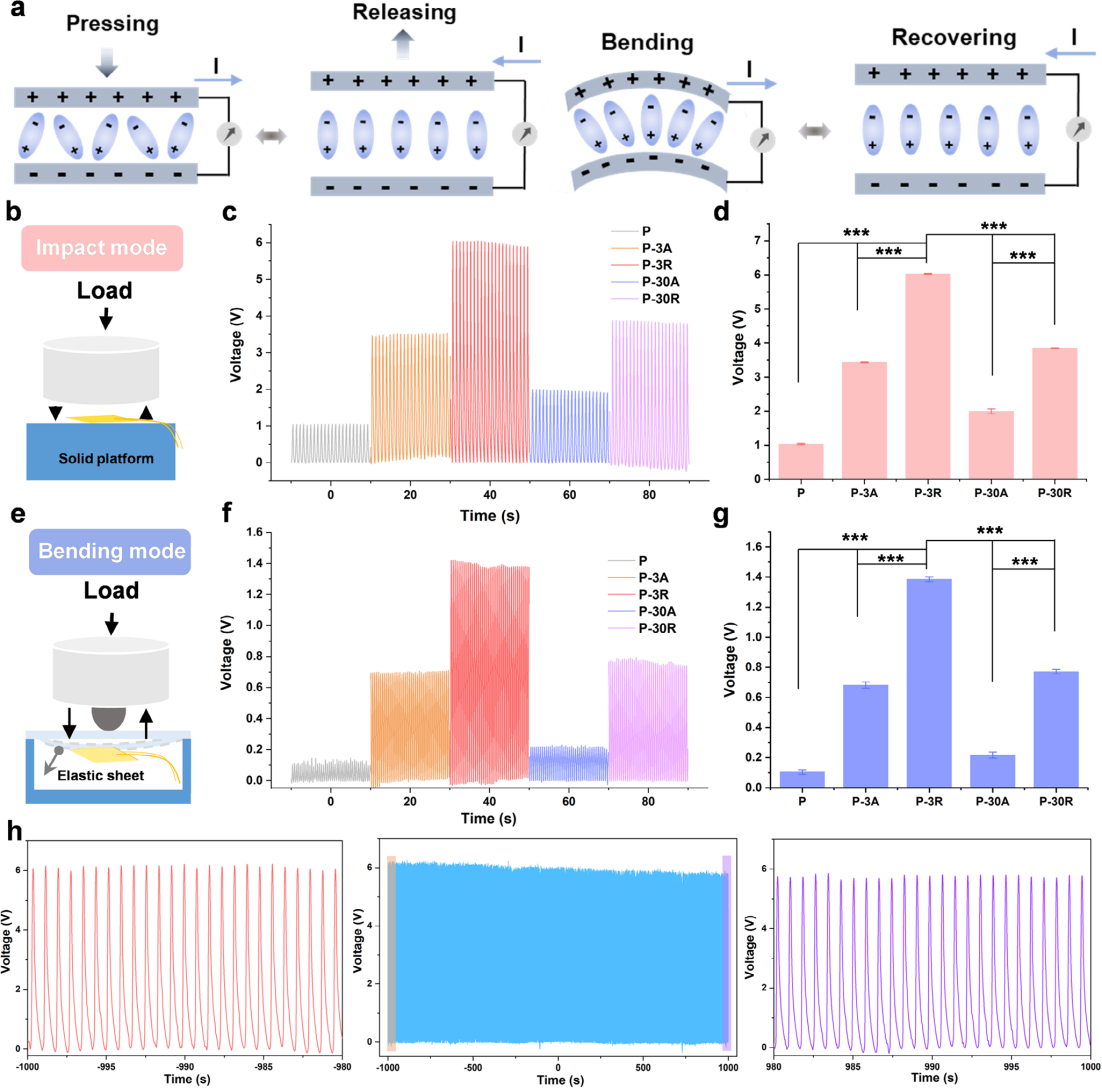
Piezoelectric properties of different fiber membranes. (a) Illustration of the working principles of the fiber membrane under pressing and bending. (b) A schematic of the impact mode test. (c) Electrical output and (d) voltage statistics chart for each group of fiber membranes under impact mode (^***^ *p* < 0.001). (e) A schematic of the bending mode test. (f) Electrical output and (g) voltage statistics chart for each group of fiber membranes under bending mode (^***^ *p* < 0.001). (h) Long‐term electrical output of P‐3R fiber devices within 2000 s.

After fabricating devices from the aforementioned fiber membranes, we tested their piezoelectric properties under impact and bending modes. Before this, we tested the piezoelectric coefficients of each group. Compared to the P group, the piezoelectric coefficient increased in the groups with lower doping concentrations, while highly doped fibers all exhibit a decrease (Figure S8). As mentioned earlier, this enhancement is primarily due to the high‐aspect‐ratio needle‐shaped HAp, which acts as a heterogeneous nucleant to promote the crystallization of PLLA. The impact mode simulates the material's electrical response to instantaneous external forces. In this mode, when the device undergoes mechanical stress, its internal charge distribution shifts, thereby generating electrical charges [37]. Similarly, the bending mode simulates the piezoelectric response of a device under periodic bending loads [38]. When a piezoelectric fiber bends, tensile and compressive stress gradients are generated within it. The piezoelectric effect redistributes internal charges, generating a voltage signal on the fiber membrane surface (Figure 4a) [16]. A cyclic impact force of 5 N was applied to each group of devices (Figure 4b), and the actual measurement platform is shown in Figure S9. Compared to the purely oriented P group (0.98 V), the HAp‐doped P‐3A, P‐3R, P‐30A, and P‐30R groups all exhibited higher output. The higher voltage of the P‐3A group compared to the P‐30A group can be attributed to the higher crystallinity of the upper PLLA‐HAp fibers in P‐3A (Figure 3g), indicating that more PLLA molecules are present in an ordered crystalline structure. When subjected to mechanical stress, these crystalline structures can more effectively reorient dipoles and separate charges, thereby generating a superior piezoelectric response. The P‐3R demonstrated the highest output (6.01 V) (Figure 4c,d), which may be related to stress concentration induced by low‐concentration doping [39]. Compared to P‐30A, which can only provide a uniform stress transfer zone, P‐30R offers a more dispersed stress concentration zone due to the random PLLA‐HAp fiber arrangement, resulting in superior electrical output (3.85 V). The current and capacity of each group also exhibit similar trends (Figure S10). This significant enhancement in piezoelectric performance broadens the threshold of traditional PLLA devices, demonstrating immense potential for monitoring applications.

To minimize the impact of air friction, we conducted the tests in the bending mode (Figure 4e). The device was placed on the back of the elastic substrate, and the elastic sheet was cycled with a displacement of 2 mm (Figure S11). Similar results are shown in Figure 4f,g, where the P‐3R group exhibited a voltage of 1.38 V compared to the 0.10 V output (P group). Interestingly, compared to P‐30A, disordered P‐30R exhibits an electrical output of 0.77 V. The difference between the two lies in the deposition of aligned PLLA‐HAp and random PLLA‐HAp on the PLLA fiber. At this stage, the high HAp content has failed to enhance the crystallinity of PLLA fibers. Therefore, we hypothesize that the increased electrical output of P‐30R stems primarily from HAp's stress‐transfer and stress‐concentration effects within the fiber membrane.

Additionally, we conducted supplementary measurements of the dielectric constant for each group of fiber membranes. As shown in Figure S12, the incorporation of HAp generally increases the dielectric constant of the composite material; however, compared to the control group (P), this increase represents only a slight change. We speculate that, in P‐3R fiber devices, the effect of structural stress concentration on piezoelectric output is more critical. Meanwhile, to further eliminate the influence of interfacial effects within the fibers, the SEM morphological images and EDS mapping data of the P‐3R group interface (Figure S13). These images clearly show that there is no obvious delamination between the upper and lower layers of fibers in the P‐3R group; instead, they exhibit good interpenetration and bonding. The distribution of HAp particles also confirms the continuity of the fibers. The mechanical tensile results showed that there are no particularly significant differences in mechanical properties between P and the P‐3R group (Figure S14). This provides direct evidence that the interlayer connections are robust enough to facilitate stress transfer. Furthermore, to rule out possible triboelectricity, capacitance, or other interfacial effects between the bilayer structures, we prepared and tested PLLA‐aligned/PLLA‐random composite fiber membranes (Pr‐Pa). The results in the impact and bending modes confirmed that their voltage outputs (∼1.2 and 0.09 V, respectively) were similar to those of the purely aligned PLLA fiber membranes (Figure S15). This further demonstrates that the enhanced piezoelectric performance of our P‐3R fiber membranes is attributable to the stress concentration effect generated by HAp, rather than interface effects resulting from the topological structure.

To verify the long‐term stability of the device, we tested the electrical output of the P‐3R device over 2,000 s. As shown in Figure 4h, under cyclic loading, the P‐3R initially exhibited an output of 6.0 V. By the end of the 2000 s cycle, the device's output decreased to 5.8 V. This slight decrease indicates the device's excellent stability while also confirming the possibility that stress concentration enhances the piezoelectric effect. This is because under frequent loading conditions, the structures within the device and the changes in force transmission are directly affected. The long‐term performance evolution of the P‐3R device was further evaluated under physiological‐like conditions in Figure S16. The piezoelectric output gradually decreases during in vitro degradation in PBS (37°C), while remaining clearly detectable in the early stage. At later stages, the formation of microcracks within the fibrous membrane disrupts the structural integrity and stress transfer pathways, leading to a decline in output. Nevertheless, the device's overall stability is sufficient for routine physiological monitoring and health management.

### 2.4 The Mechanism of Stress Concentration Enhancing the Piezoelectric Effect

To better understand the effect of HAp on the piezoelectric output of PLLA fibers, we performed piezoelectric force microscopy (PFM) phase imaging, Kelvin probe force microscopy (KPFM) surface potential mapping, and local Young's modulus measurements on P and P‐3R composite fiber membranes (Figure S17). Compared to the P group, which exhibited a relatively uniform Young's modulus, the P‐3R group demonstrated a more heterogeneous distribution of Young's modulus. This heterogeneity in local mechanical properties is a key prerequisite for the formation of stress gradients and helps explain the occurrence of stress concentration phenomena. The KPFM maps of the P‐3R group clearly reveal significantly higher local surface potentials. In the P‐3R group, significantly enhanced and locally concentrated signals can be observed at multiple locations, indicating that the material exhibits stronger piezoelectricity at these microscopic locations. Raman spectroscopy was also performed on fibers in the P and P‐3R groups before and after the application and removal of tensile stress (Figure S18). Specifically, the peak at 870 cm^−1^—corresponding to the crystalline phase of PLLA—exhibited significant enhancement in both the P and P‐3R groups after tensile loading. In the 1000–1150 cm^−1^ range, we observed greater peak shifts in the P‐3R group after stretching. These peaks are associated with ester (C─O─C) and backbone vibrations, reflecting changes in molecular chain orientation, conformation, and internal stress states induced by stretching. This indicates the presence of greater local strain and possible plastic deformation within the P‐3R fibers.

**FIGURE 5:**
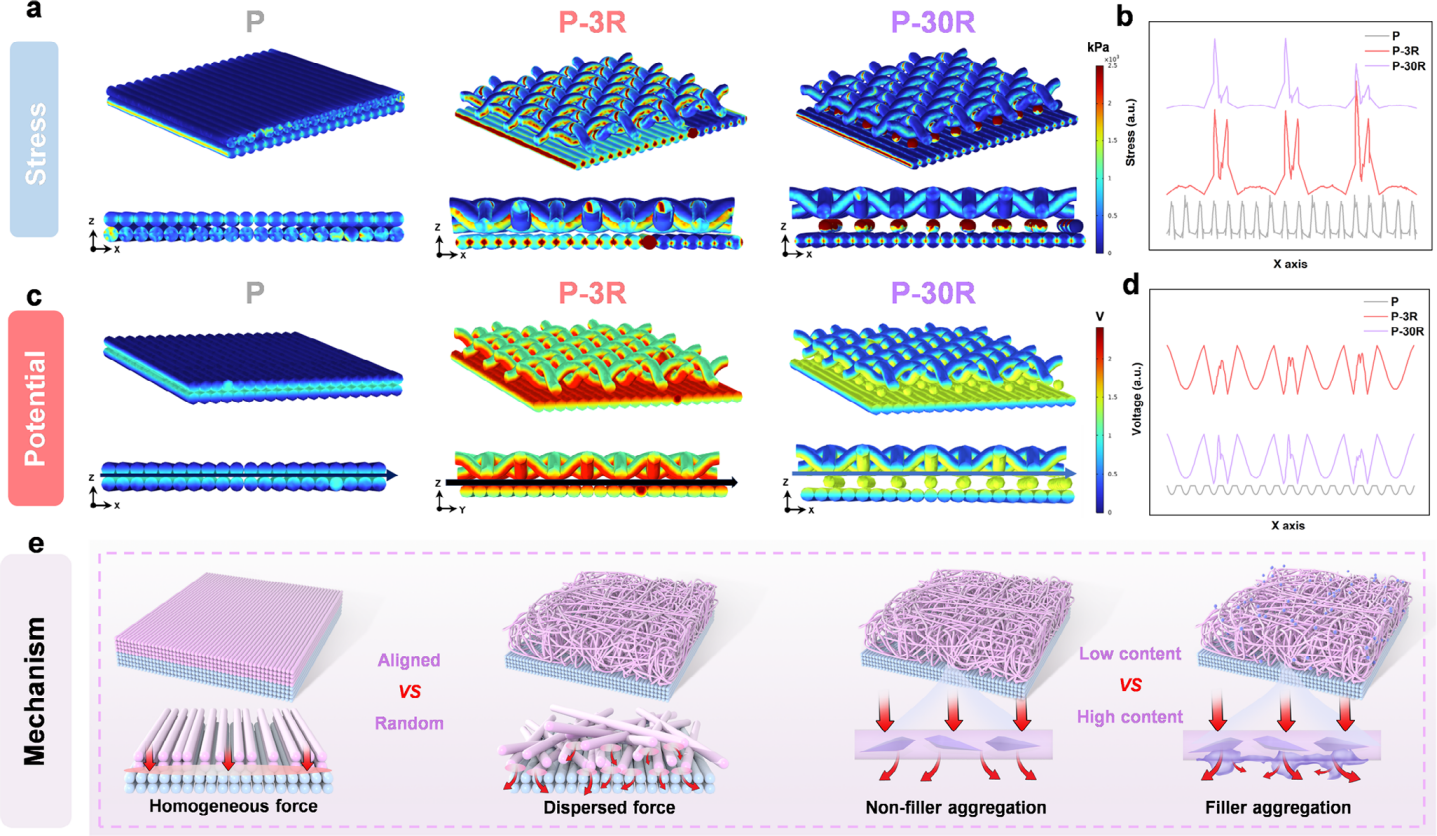
Mechanism of stress concentration enhancing piezoelectric performance. (a) Stress top view and side view of P, P‐3R, and P‐30R fiber membranes in COMSOL analysis. (b) Stress distribution along the *x*‐axis of P, P‐3R, and P‐30R fiber membranes. (c) Potential of P, P‐3R, and P‐30R fiber membranes in COMSOL analysis. (d) Potential distribution along the *X*‐axis of P, P‐3R, and P‐30R fiber membranes. (e) The effect of aligned/random PLLA‐HAp fibers and low‐concentration/high‐concentration HAp doping on the overall piezoelectricity of fiber membranes.

Subsequently, COMSOL simulation was employed to compare and analyze the stress and electric potential field of three groups of piezoelectric fibers with different topologies (P, P‐3R, P‐30R) under the combined effects of applied force and interfacial coupling. The simulation inherently assumes the strong coupling achieved through our fabrication process to investigate the effects of the topology on stress distribution, and it did not individually introduce adjustable interlayer bonding strength or friction coefficients, nor did it establish explicit frictional/sliding contact models. This analysis aimed to reveal the influence of stress concentration effects on piezoelectric performance. Figure 5a shows that under identical external force loading, the strain distribution in the P group (parallel alignment) remains relatively uniform, primarily concentrated on the fiber surface. Due to its simple structure, stress transfer pathways are simpler, resulting in smaller strain gradients at the surface and ends. Displacement responses along the x‐direction exhibit good synchrony. In contrast, the strain distribution in the P‐3R group is markedly more complex. Significant strain concentration occurs in the fiber interface (Figure 5b; Figure S19). Both the top view and cross‐section reveal that strain no longer exhibits a simple uniform distribution but instead displays periodic fluctuations and localized high‐strain zones. This suggests that the introduction of disordered fibers in P‐3R alters stress transmission pathways, allowing for a more efficient distribution and transfer of stress both within and between fibers. Although the P‐30R group is similar to the P‐3R, the most pronounced strain concentration occurs in the agglomerated regions of HAp. This stress distribution may fail to effectively transmit stress to the underlying ordered PLLA fibers due to stress mismatch within the inorganic‐organic interfaces.

Simultaneously, the simulated potential distribution exhibits patterns consistent with the strain distribution. This is because the potential distribution is closely related to regions of concentrated strain, where high strain areas often correspond to high potential values. The potential distribution of the P‐structure aligns with the stress distribution, exhibiting low and uniform potentials with gentle periodic fluctuations (Figure 5c,d). The potential distributions of the P‐3R and P‐30R groups exhibit elevated potential values in numerous regions. As seen from the top view and cross‐section, the potential generates distinct localized high‐potential regions within the fiber‐interwoven area. However, the P‐3R structure demonstrates more pronounced potential fluctuations and higher peak potentials, indicating superior efficiency and potential for converting mechanical energy into electrical energy.

It is worth noting that we emphasize the distinct advantages of combining random low‐doped fiber membranes with aligned fiber membranes (Figure 5e). Compared to pure PLLA fibers, it provides an excellent stress‐concentration effect. Compared to fully aligned composite fiber membranes (P‐3A group), the P‐3R group also offers stress‐concentration effects and achieves a more dispersed stress distribution. Compared to the highly doped P‐30R, P‐3R avoids filler agglomeration, thereby ensuring effective stress transfer. Although some studies have reported that stress concentration can amplify the piezoelectric effect, most of these involve piezoelectric fillers such as barium titanate and zinc oxide [18, 40]. In contrast, HAp, as a non‐piezoelectric filler, also exhibits stress concentration effects that enhance the piezoelectric properties of fibers. This structural enhancement effectively translates into improved macroscopic energy conversion efficiency, allowing our PLLA system to achieve a normalized voltage density comparable to various state‐of‐the‐art piezoelectric biomaterials, as detailed in Table S1. It is also worth considering that some technologies, such as AI‐based reverse‐engineering and topological optimization algorithms, may further optimize device fabrication, striving to achieve a better balance among multiple key factors—including stress concentration, effective stress transfer, filler dispersion, doping ratios, device stability, and manufacturability—thereby yielding superior piezoelectric performance.

### 2.5 Personalized Physiologic Signal Monitoring Platform Based on Piezoelectric Fibers

**FIGURE 6:**
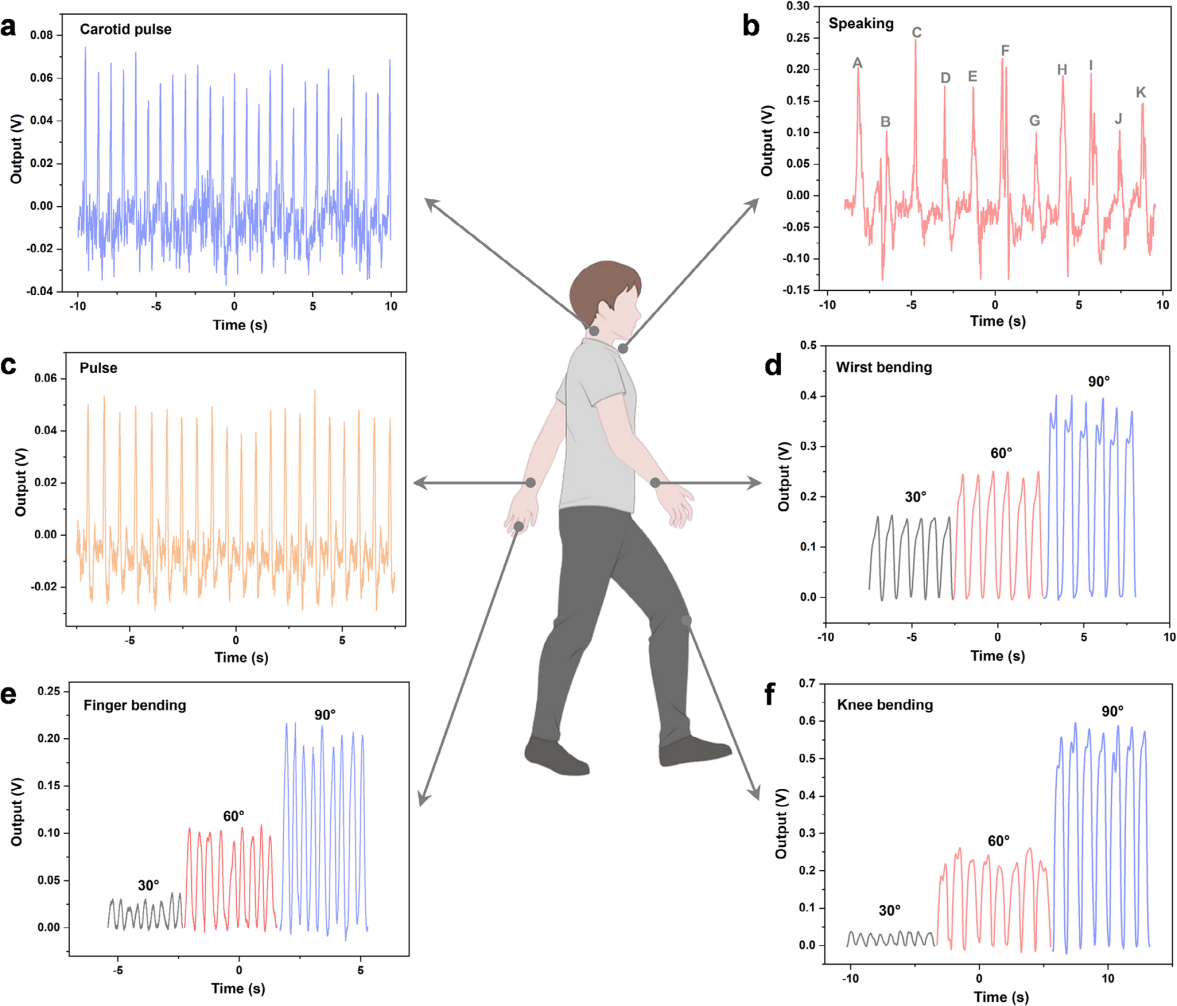
Physiological activity monitoring using the P‐3R fiber device. (a) Monitoring of the carotid pulse signal. (b) Monitoring the Adam's apple region during the pronunciation of different words. (c) Monitoring of pulse signals. Monitoring of flexion movements in the (d) wrist joints, (e) finger joints, and (f) knee joints.

Based on the outstanding piezoelectric properties and flexibility of the P‐3R device described above (Figure S20), we have incorporated it into a personal health monitoring platform. As shown in Figure 6a,c, signals from the carotid and radial arteries were successfully acquired, exhibiting the characteristic, distinct rhythmic patterns of arterial pulsations [41]. This demonstrates the high sensitivity of piezoelectric fibers to minute mechanical deformations induced by blood flow.

Furthermore, the platform excels in quantifying physical activity. Figure 6b displays electrical signal outputs generated during vocalization. Distinct peaks labeled A through K correspond to individual phonemes or vocal units, indicating the system's ability to capture intricate vibrational dynamics of the vocal cords and surrounding tissues. Voltage outputs generated by different degrees of wrist, finger, and knee joint movement (30°, 60°, 90°) are captured (Figure 6d–f). The increasing amplitude of piezoelectric signals with greater bending angles further validates the platform's capability to quantify joint range of motion and track movement patterns, highlighting the system's versatility in monitoring fine motor skills. This device demonstrates the ability to detect and quantify multiple physiological signals, including cardiovascular pulses, vocalizations, and joint movements across varying amplitudes. This highlights its significant application potential in continuous health tracking, athletic performance analysis, and rehabilitation monitoring.

### 2.6 Monitoring of Knee Joint Movement and Cardiac Activity in Pigs

**FIGURE 7:**
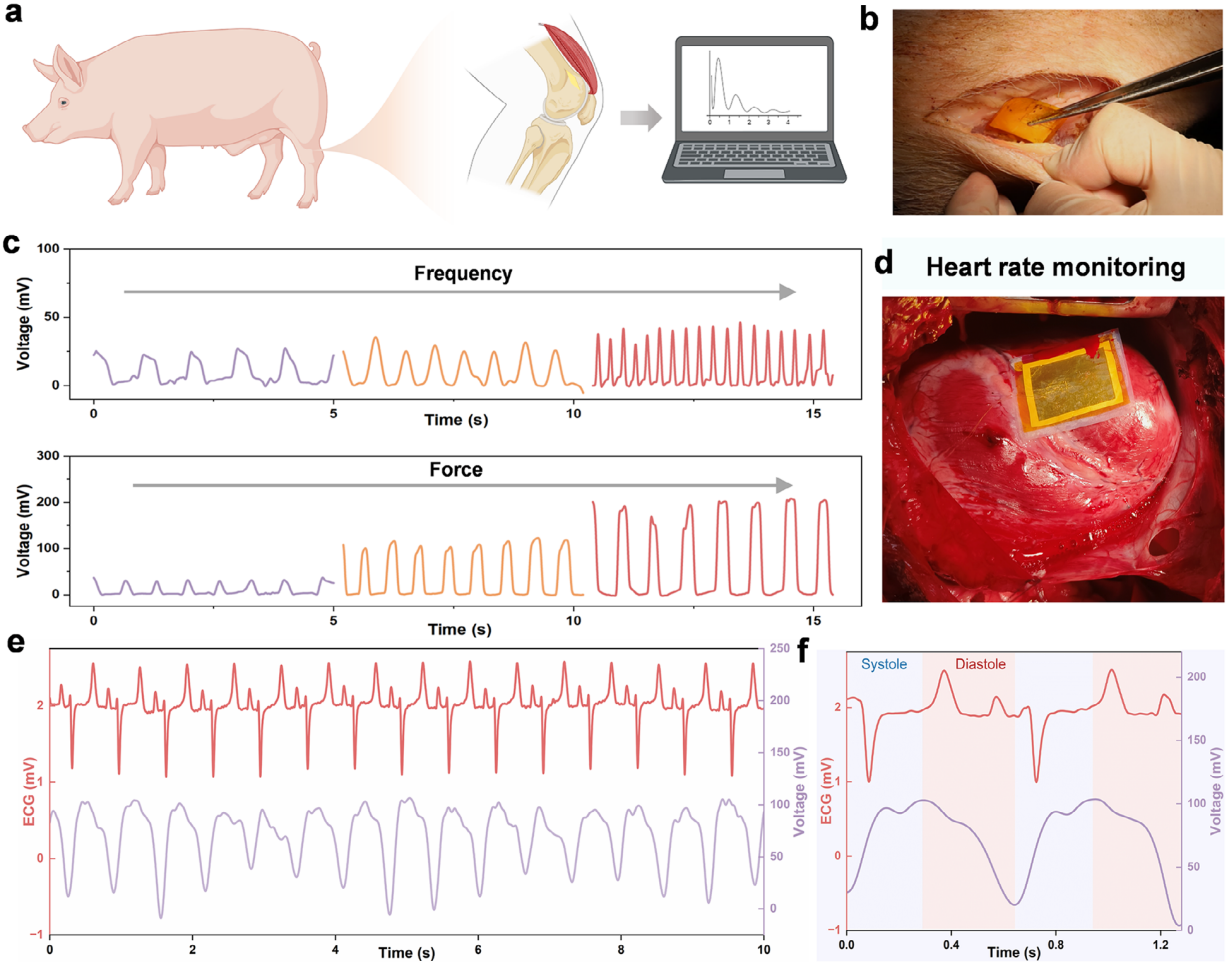
Real‐time monitoring of knee joint and cardiac activity in pigs. (a) Schematic diagram of knee joint monitoring in pigs. (b) Digital photograph of the device implanted in the knee joint area. (c) Real‐time signals when forces of varying frequencies and magnitudes are applied. (d) Digital photograph of the device adhering to the heart's surface. (e) The ECG signal recorded during heartbeats and the device output, and (f) its amplification graph.

To evaluate the performance of this piezoelectric fiber platform in physiological monitoring, we validated it on pig knee joint movements (Figure 7a,b) and cardiac activity. Figure 7c demonstrates the device's response to different frequencies and mechanical stimuli applied to the pig knee joint. The results show a trend where electrical output increases as the applied movement frequency rises. This corresponds to the periodic flexion and extension of the knee joint, with higher‐frequency movements generating denser electrical signals. Similarly, the voltage signal intensifies as the applied force increases. This direct relationship between mechanical force and electrical output validates the fiber's piezoelectric properties and its ability to quantify applied stress. This confirms the piezoelectric fiber membrane's potential for precise detection of diverse joint movements.

Additionally, Figure 7d demonstrates the platform's capability to detect cardiac activity. By comparing standard ECG signals (red) with voltage signals (purple) generated by the piezoelectric fiber devices placed on the epicardial surface of the left ventricle, we observed consistent rhythmicity between the two physiological signals across the monitoring period (Figure 7e). Figure S21 shows simultaneous recording of ECG and piezoelectric signals over a 100 s period, which demonstrates stable signal acquisition and consistent temporal correspondence between the two modalities. The quantitative comparison of heart rate, presented in Figure S22, further confirms the high consistency between the heart rates derived from the piezoelectric device and the ECG gold standard. A low‐frequency fluctuation attributed to respiration‐induced cardiac motion is present in the raw signal and was reduced by applying a 0.5 Hz high‐pass filter (Figure S23). Further detailed waveform analysis in Figure 7f reveals that the piezoelectric signal rises synchronously with the QRS complex during ventricular systole and decays during the T wave in ventricular diastole. This indicates that the device can output characteristic signals that capture the full‐cycle mechanical responses of the left ventricle, demonstrating its significant potential for clinical research applications.

## 3 Conclusion

In summary, inspired by bone piezoelectricity and building upon prior research on HAp's role as a nucleating agent, this work investigates multiple enhancement pathways for PLLA electrospun fibers using non‐piezoelectric inorganic filler HAp. We further confirm and expand upon findings that HAp with a high aspect ratio, when doped into electrospun PLLA fibers at low concentrations, serves as a heterogeneous nucleation site, significantly improving the crystallization process of PLLA and thereby enhancing its piezoelectric response. Crucially, the optimized P‐3R device significantly enhances the piezoelectric output performance of conventional PLLA devices. This improvement stems from stress concentration effects when disordered, low‐concentration HAp‐doped PLLA fiber membranes serve as the upper layer, enabling effective stress transfer to the underlying PLLA fiber. This platform endows the real‐time monitoring of human physiological activities, knee joint movements, and cardiac activity in pigs, demonstrating immense potential for clinical applications. Most importantly, this simple membrane integrates non‐piezoelectric fillers, a topological fiber structure, and stress concentration effects to achieve significant piezoelectric amplification, offering an alternative approach for designing next‐generation high‐performance piezoelectric devices.

## 4 Experimental Sections

### 4.1 Materials and Chemicals

PLLA and PLA were obtained from Esunmed. (Shenzhen, China). Hydroxylapatite (HAp) was purchased from Macklin Biochemical Technology Co., Ltd. (Shanghai, China), where HAp exhibits various morphologies, including nanoscale, needle‐like, and rod‐like shapes. Hexafluoroisopropanol (HFP, 99.5%) was purchased from Aladdin Biochemical Technology Co., Ltd. (Shanghai, China). The phosphate‐buffered solution (PBS) was purchased from Solarbio Science & Technology Co., Ltd.

### 4.2 Fabrication and Characterization of PLLA‐HAP Nanofiber Membrane

First, a basic electrospinning solution was prepared by dissolving PLLA (10 w/v%) in HFP. Subsequently, HAp‐Nano, HAp‐Needle, and HAp‐Rod were incorporated into the spinning solution to investigate the effects of different HAp morphologies on the crystallization ability and piezoelectric properties of PLLA electrospun fiber membranes. The solution was electrospun at a flow rate of 1 mL/h under a voltage of 14 kV, with a tip‐to‐collector distance of 15 cm. The morphologies of the PLLA‐HAp membranes were characterized by scanning electron microscopy (SEM, SU8020, Japan), and the crystalline structure of the nanofiber membranes in each group was determined using an x‐ray diffractometer (XRD, PANalytical).

### 4.3 Fabrication and Characterization of Composite Nanofiber Membrane

Similar to the procedure aforementioned, 3 or 30 wt.% hydroxyapatite (relative to PLLA mass) was sequentially added to prepare PLLA‐HAp (3 wt.%) and PLLA‐HAp (30 wt.%) spinning solutions. By adjusting solution and collector rotation speeds, five sets of fiber membranes were obtained: the aligned nanofiber membrane (PLLA) was produced by spinning on a rotating drum collector at 2000 rpm for 2 h. Based on the aligned PLLA nanofiber membrane obtained at 2000 rpm for 1 h, the rotational speed was sequentially adjusted to 2000 and 200 rpm for 1 h to obtain aligned/random‐oriented PLLA‐HAp (3 wt.%) nanofiber membranes (P‐3A, P‐3R). The same method was applied to a 30 wt.% PLLA‐HAp solution to similarly obtain aligned/randomly oriented PLLA‐HAp (30 wt.%) nanofiber membranes (P‐30A, P‐30R). All membranes were subsequently annealed at 150°C for 12 h in an oven, ultimately forming piezoelectric membranes.

The morphologies of the piezoelectric membranes were characterized by SEM, and elemental mapping was performed to verify the HAp content and distribution within the composite nanofibers. Fourier‐transform infrared (FTIR) spectroscopy (Nicolet 8700, Bruker) was used to analyze the changes in the chemical composition of the piezoelectric nanofiber membranes in each group. The crystalline structure of the piezoelectric nanofiber membranes in each group was determined using XRD. The tensile properties of the scaffolds were evaluated using an ESM301/Mark‐10 tensile testing system. The crystallization ability of each fiber group was tested using a differential scanning calorimeter (DSC). Under a nitrogen atmosphere, the heating rate was set to 10°C/min, and the test was conducted at temperatures ranging from 25°C to 250°C. The calculation of crystallinity was based on previous work [42]. The Raman spectrometer (LabRAM HR Evolution) was used to analyze changes in spectral peaks in piezoelectric fiber membranes before and after stretching.

### 4.4 Piezoelectric Properties of Composite Nanofiber Membranes

A piezoelectric constant measuring instrument (ZJ‐6BN) was used to test the piezoelectric coefficients of each group of fiber membranes. Piezoelectric force microscopy (PFM) phase imaging, Kelvin probe force microscopy (KPFM) surface potential mapping, and local Young's modulus measurements were performed using an atomic force microscope (AFM) (Bruker Dimension Icon). Each group of fiber membranes was cut into 1.5 × 1.5 cm rectangles and coated with conductive aluminum tape on both sides to serve as electrodes. Copper wires were connected to the electrodes on opposite sides, and the entire assembly was sealed with polyimide (PI, DuPont) tape to form a piezoelectric sensor. The output electrical signal was measured directly using an electrometer (Keithley 6517) and an oscilloscope (HDO 6104). The voltage output, current, and charge were measured for each group under a 5 N impact force. Subsequently, the magnitude of the impact force was varied to record the device's voltage output, and the long‐term stability of the voltage output over 2000 s was documented.

To minimize the effect of friction on device output, we also tested the output of each device group under bending conditions. Specifically, the devices were placed on the back of an elastic sheet, secured with PI tape of the same material, and the voltage output was recorded during the elastic sheet's bending and recovery cycles. The piezoelectric performance of each group was then measured and compared.

### 4.5 COMSOL Analysis of the Piezoelectric Enhancement Mechanism

Using the 3D Simulation Module in COMSOL software, the model was optimized to investigate the piezoelectric effect of composite membranes at different doping concentrations (3 and 30 wt.%). This case employed COMSOL software for the entire simulation mesh, comprising a total of 46 540 domain elements, 5922 boundary elements, and 328 edge elements. All piezoelectric‐mechanical simulations were performed using COMSOL Multiphysics 6.2.

### 4.6 Monitoring of Human Physiological Signals

To evaluate the optimized device's sensitivity to weak forces and physiological activities, some common physiological activities were used as models for testing. The device was attached to the volunteer's wrist and carotid artery to read pulse conditions, attached to the Adam's apple to monitor pitch variations when the volunteer read aloud “A, B, C, D, E, F, G, H, I, J, K” and attached to the volunteer's index finger, wrist, and knee to monitor voltage output at different flexion levels of the test sites.

### 4.7 Leg Movement and Cardiac Monitoring in Pig Models

In the pig model, the device was placed at the knee joint of the front leg [43]. Sensory perception of the implanted device was measured by varying the frequency and intensity of movement. Subsequently, the device was secured to the heart surface to record signals of heartbeats detected by the device, which were then compared and analyzed for differences with electrocardiogram (ECG) signals [44]. All experimental procedures were strictly for the care and use of laboratory animals. The study protocol was approved by the State Key Laboratory of Cardiovascular Disease and Fuwai Hospital (ethical approval number: BJ2025‐05004).

### 4.8 Statistical Analysis

All statistical analyses were performed using Origin 2019. All data are presented as the means ± standard error of the means (S.E.M.) from at least three independent experiments. For comparisons between the two groups, a t‐test was employed. For comparisons involving more than two groups, one‐way analysis of variance (ANOVA) was used. The level of significance was indicated as: *p < 0.05, **p < 0.01, and ***p < 0.001.

## Conflicts of Interest

The authors declare no conflict of interest.

## Supporting information




**Supporting file**: advs75619‐sup‐0001‐SuppMat.docx

## Data Availability

The data that support the findings of this study are available from the corresponding author upon reasonable request.
