# Supplementary material for: Topologically Structured PLLA Fibers With Stress Concentration Effects for Health Monitoring
Source: Adv Sci (Weinh). 2026 May 11;13(43):e75619. doi: 10.1002/advs.75619 (PMC13335806; doi:10.1002/advs.75619)
Supplement: Supplementary file 1 — Supporting File: advs75619‐sup‐0001‐SuppMat.docx. [file ADVS-13-e75619-s001.docx]

***Supporting Information***

**Topologically Structured PLLA Fibers with Stress Concentration Effects for Health Monitoring**

Longfei Li^1,2,3#^, Juwei Yang^1,4#^, Yiqian Wang^1^, Qiao Yu^1^, Zhenmin Fan^1,3^, Chang Zhu^1,2^, Ming Yin^1^, Wei Hua^4^, Zhou Li^3,5^*

^1^ Beijing Institute of Nanoenergy and Nanosystems, Chinese Academy of Sciences, Beijing 101400, China.

^2^ School of Nanoscience and Engineering, University of Chinese Academy of Sciences, Beijing 100049, China.

^3^ Vita Tech Innovation Center, Tsinghua Changgung Hospital, School of Clinical Medicine, Tsinghua University, Beijing, 100084, China.

^4^ National Center for Cardiovascular Diseases, Fuwai Hospital, Chinese Academy of Medical Sciences and Peking Union Medical College, Beijing 100037, China.

^5^ School of Biomedical Engineering, Tsinghua University, Beijing 100084, China.

* Corresponding author: Zhou Li

**Email:** li_zhou@tsinghua.edu.cn (Z. Li)

#These authors contributed equally to the work.


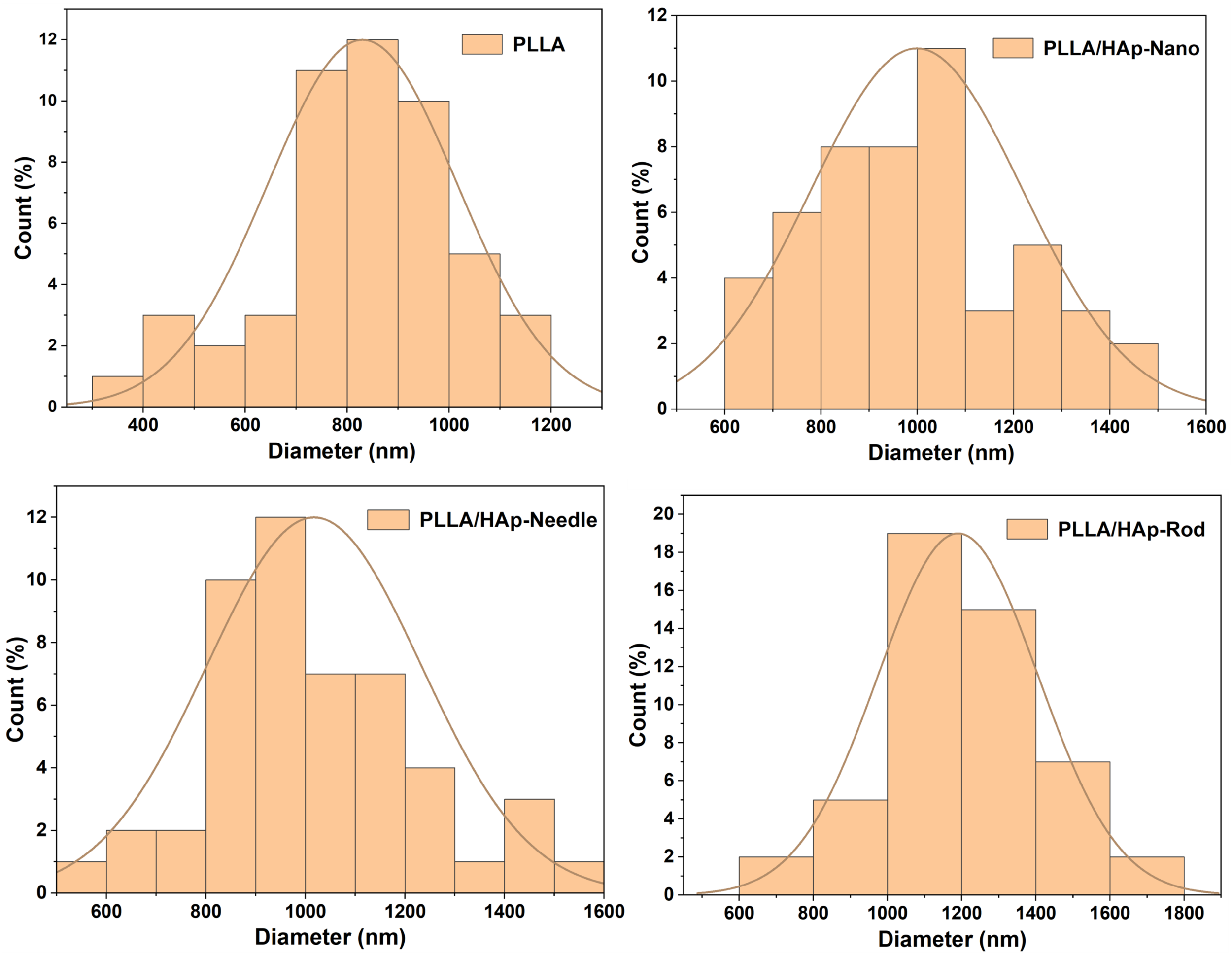


**Figure S1**. Statistical analysis of diameter distribution for PLLA, PLLA/HAp-Nano, PLLA/HAp-Needle, and PLLA/HAp-Rod fibers.


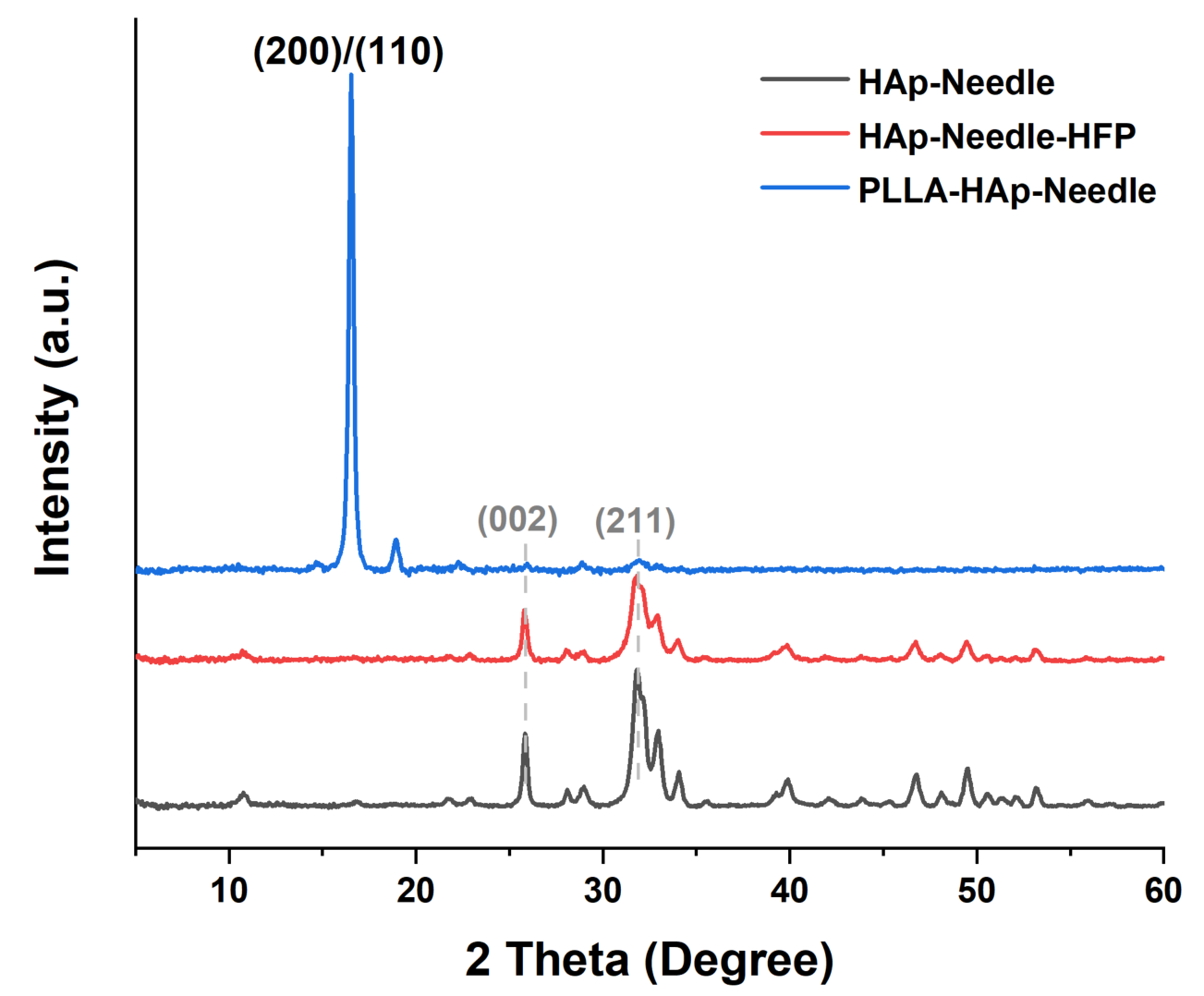


**Figure S2**. XRD curve of HAp (powder), HAp powder dissolved in HFP (HAp-HFP), and P-3R fibers.


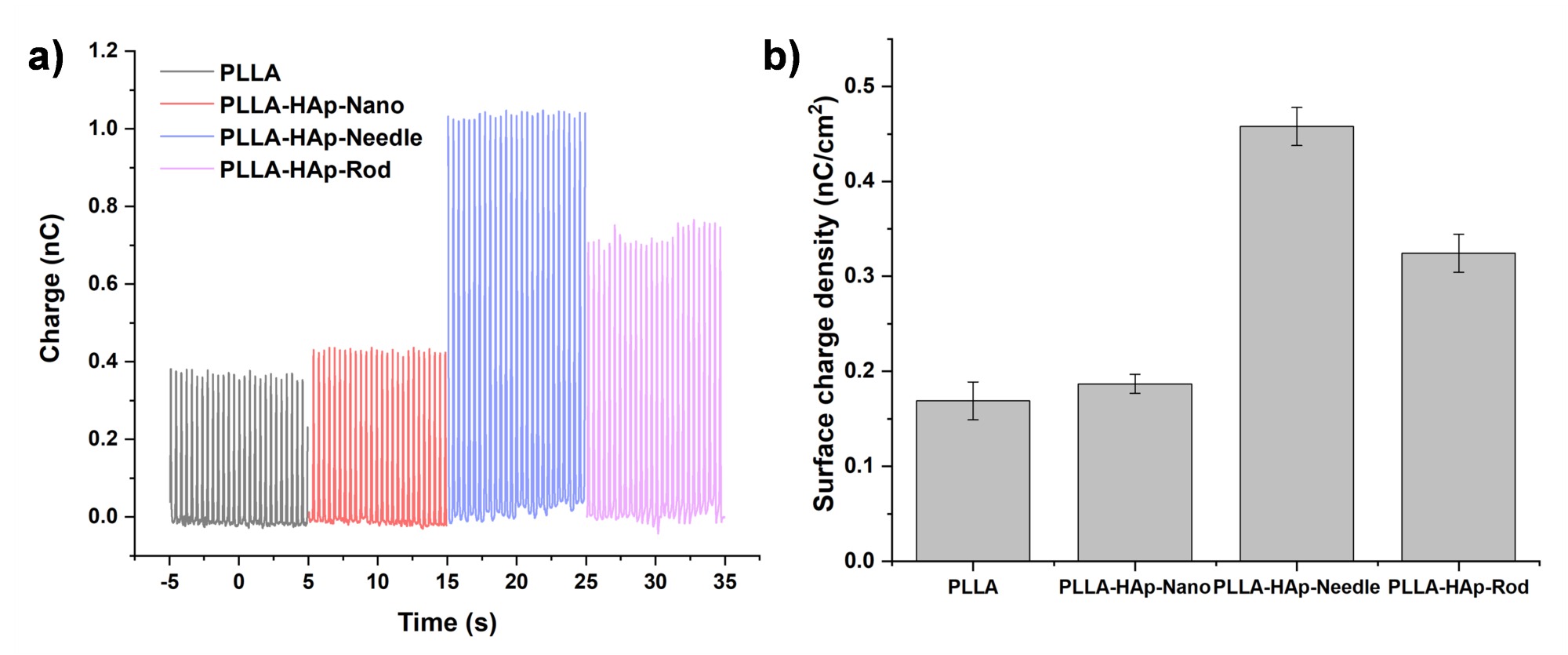


**Figure S3**. a) Charge and b) surface charge density collected from different fibers.


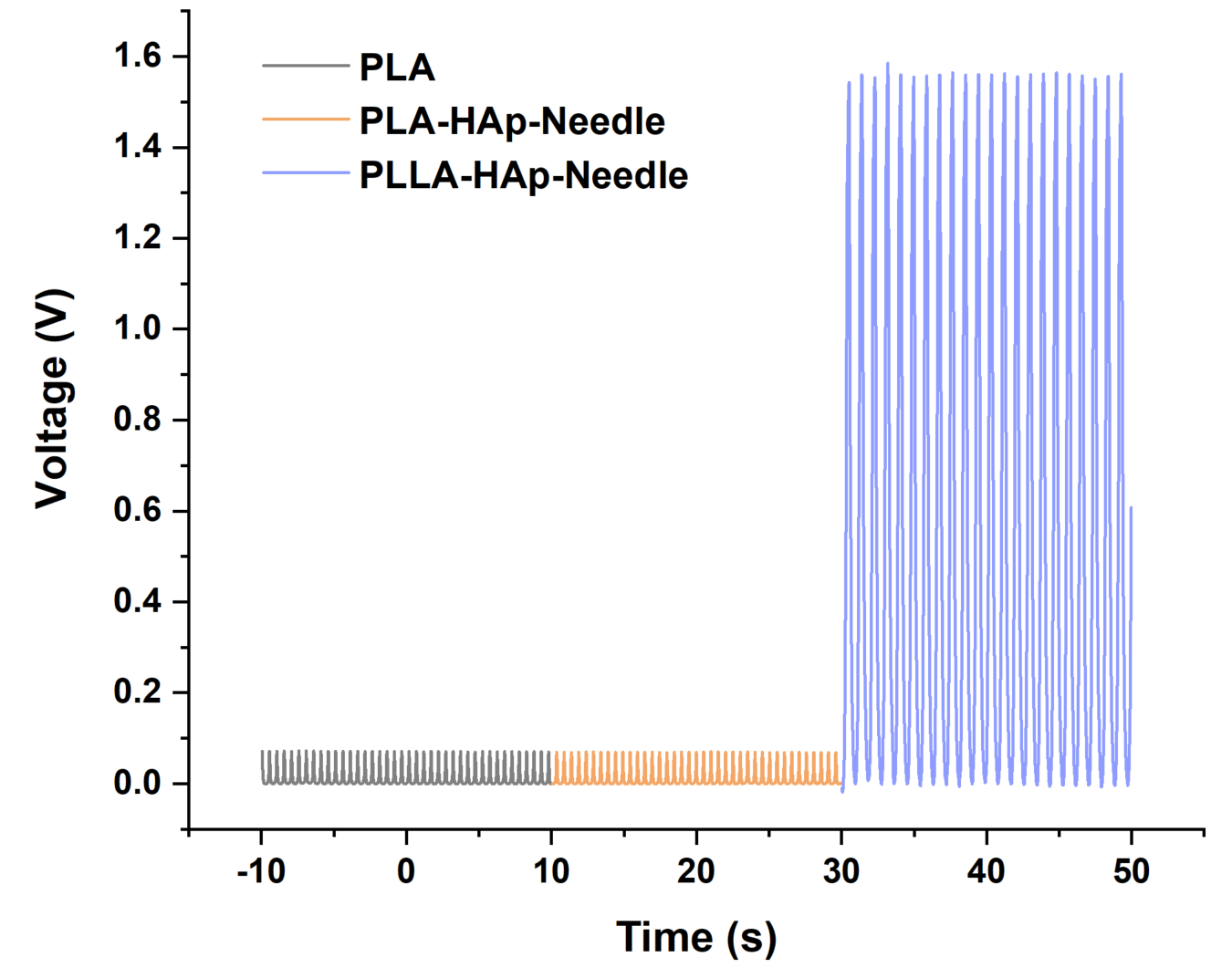


**Figure S4**. Piezoelectric output of PLA, PLA-HAp-Needle, and PLLA-HAp-Needle fibers.


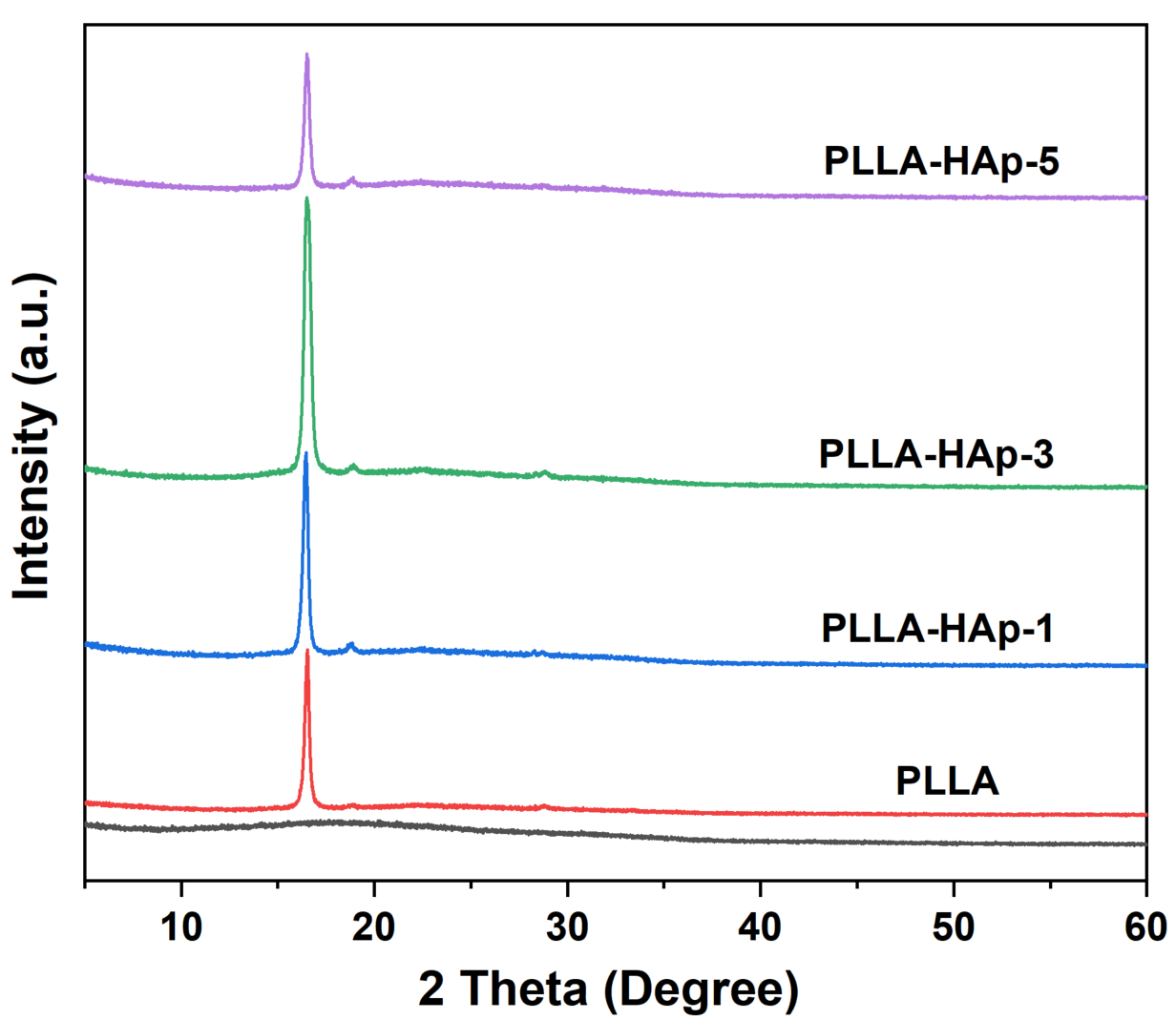


**Figure S5**. XRD curves of PLLA fibers doped with needle-like HAp at different concentrations.


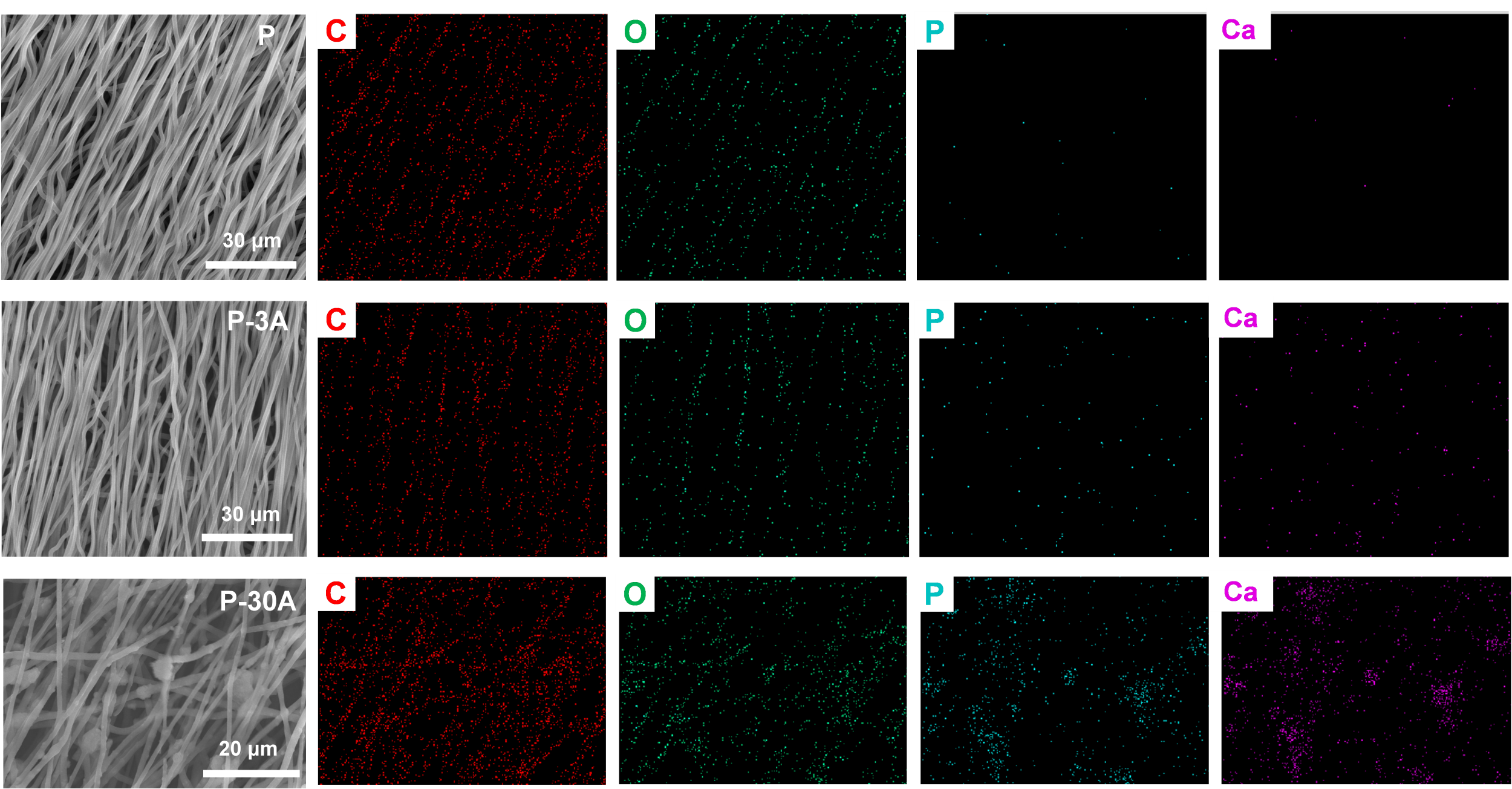


**Figure S6**. Elemental mapping distribution of P, P-3A, and P-30A fibers (C, O, P, Ca).


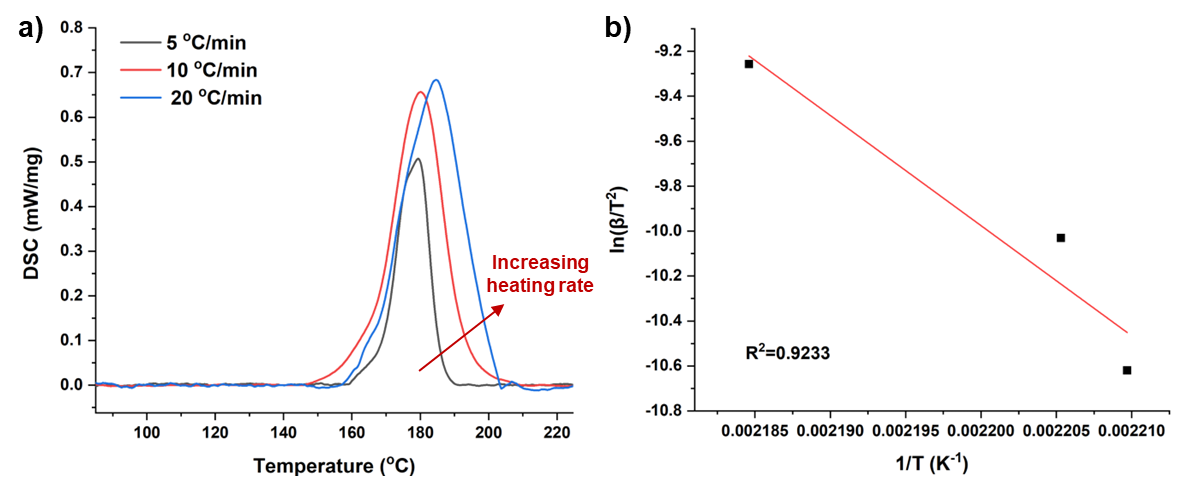


**Figure S7**. a) DSC curves of P-3R fiber at different heating rates and b) analysis of crystallization kinetics.


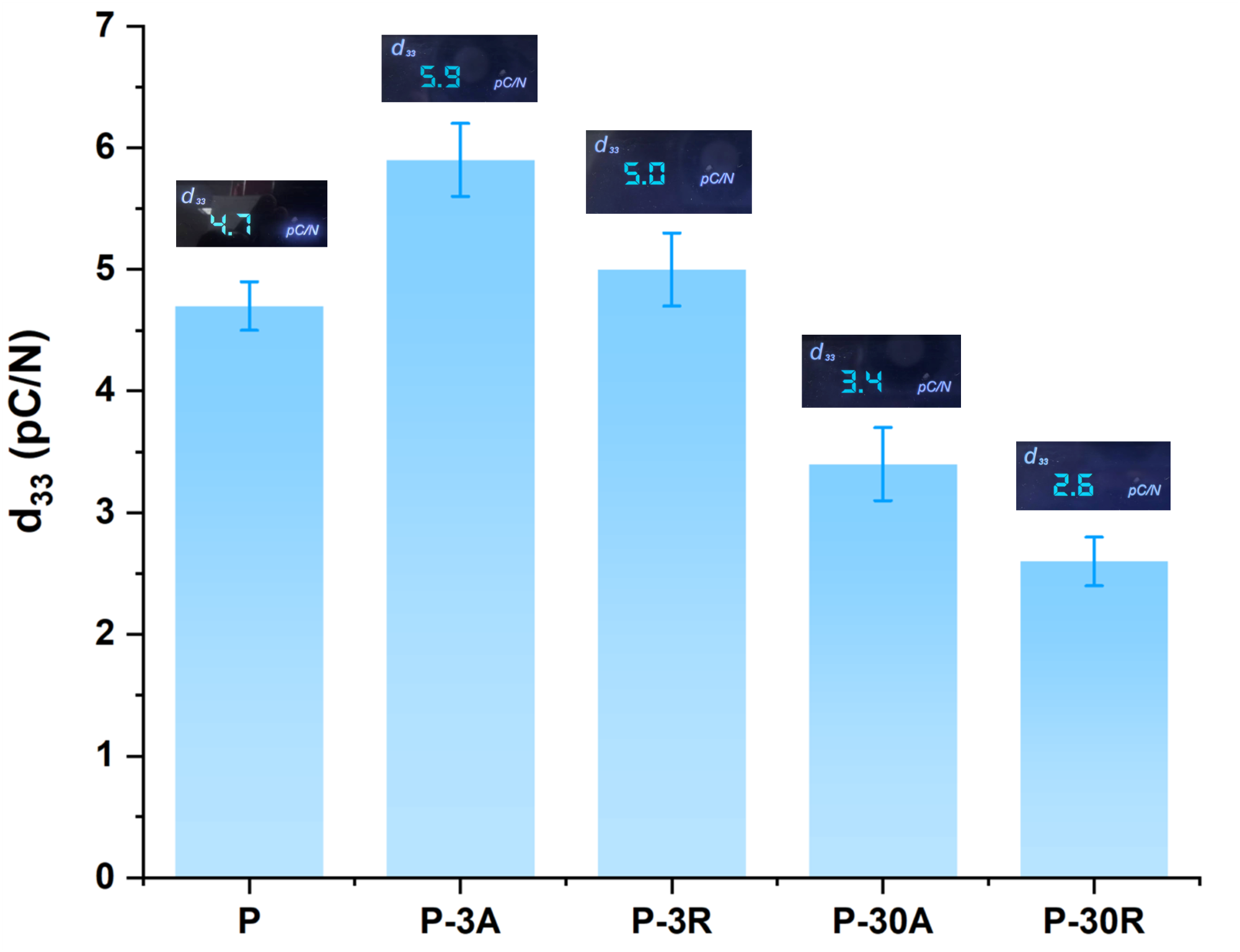


**Figure S8**. Piezoelectric coefficients of different fiber membranes.


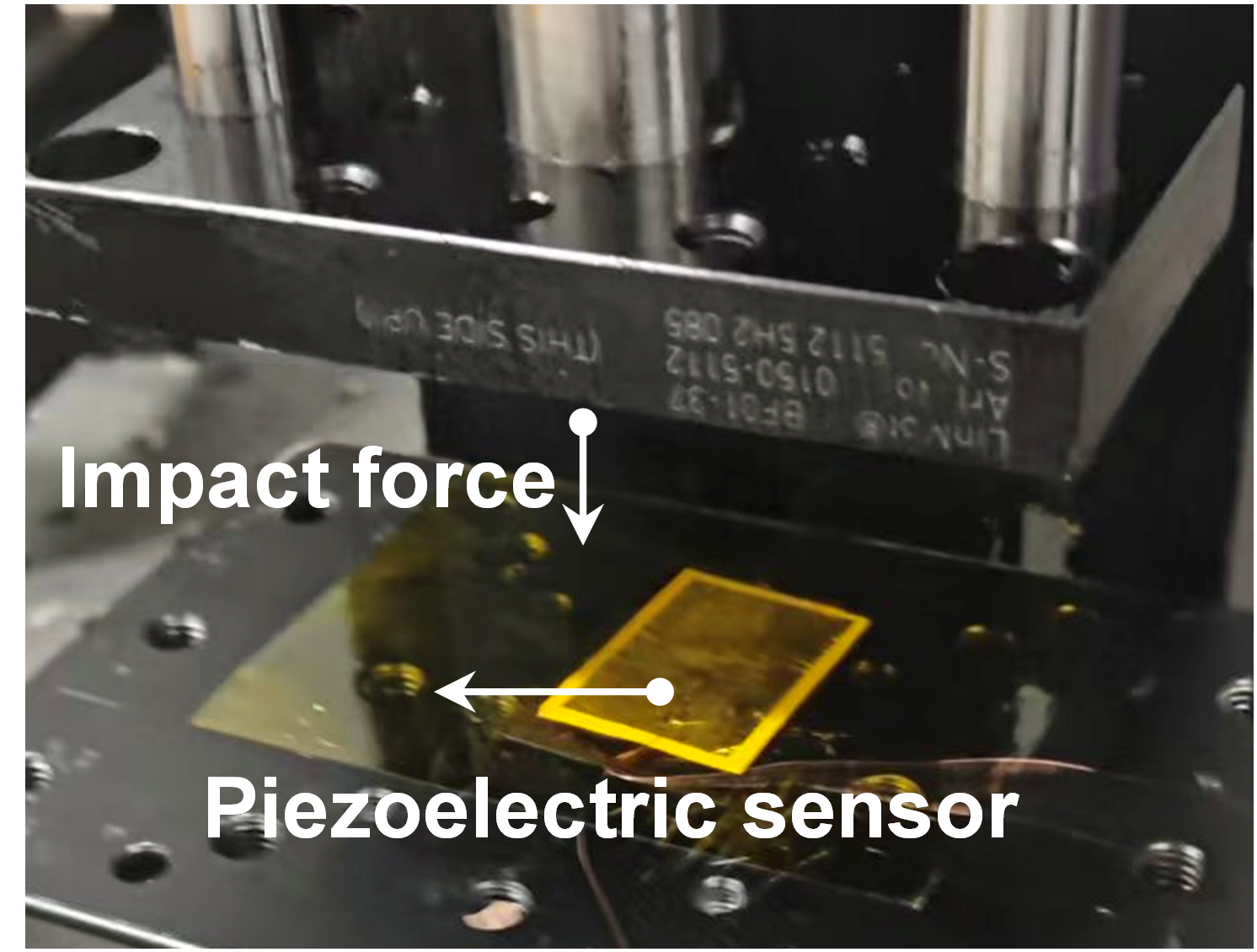


**Figure S9**. A digital photograph for impact mode testing.


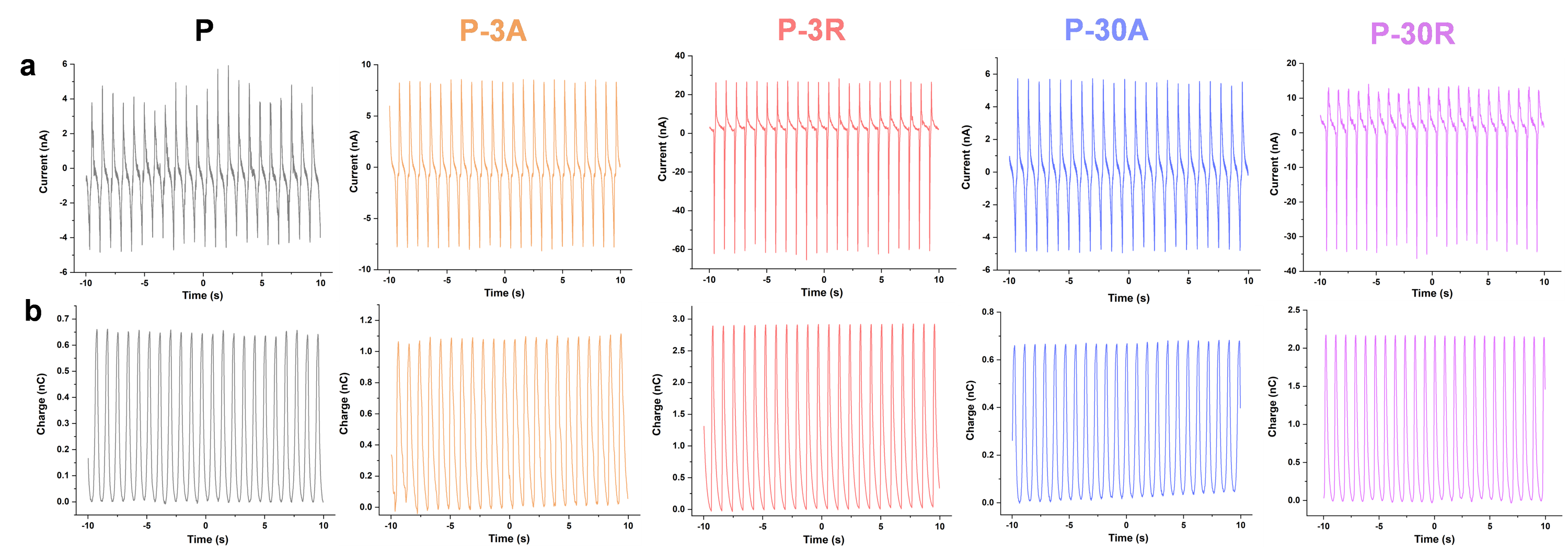


**Figure S10**. a) Current and b) charge collected from each group of fiber devices in impact mode.


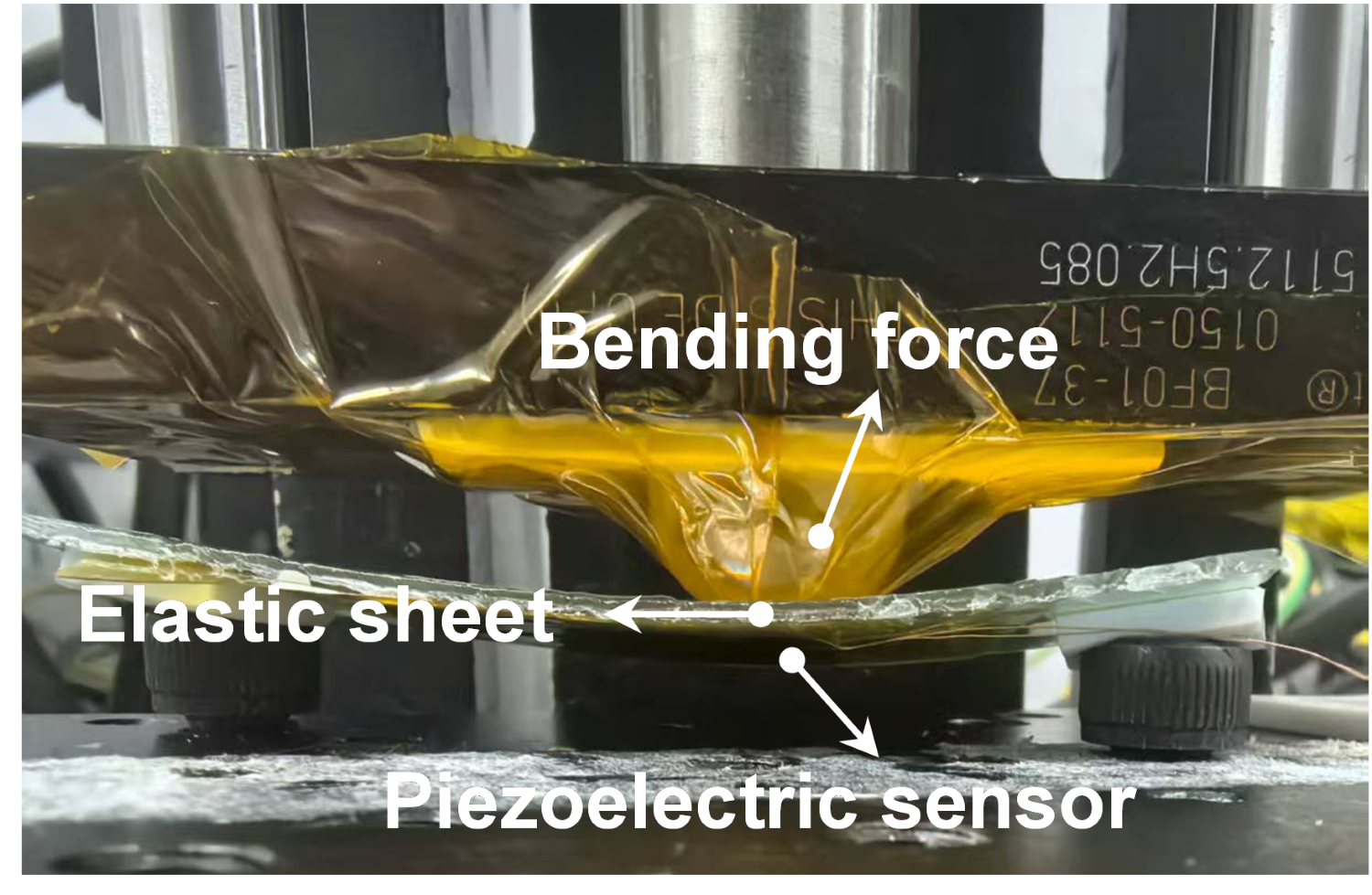


**Figure S11**. A digital photograph for bending mode testing.


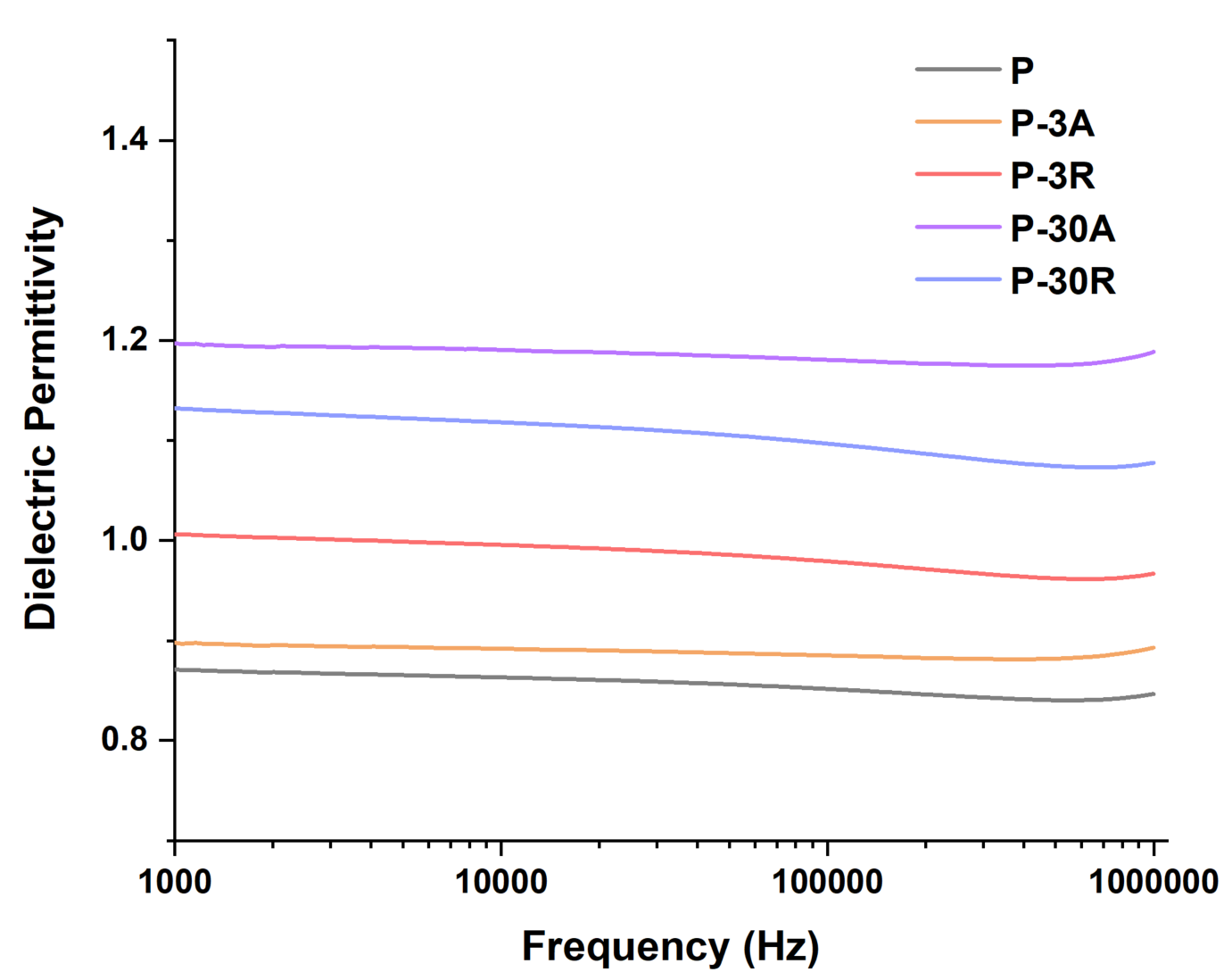


**Figure S12**. Dielectric constant of different fiber membranes.


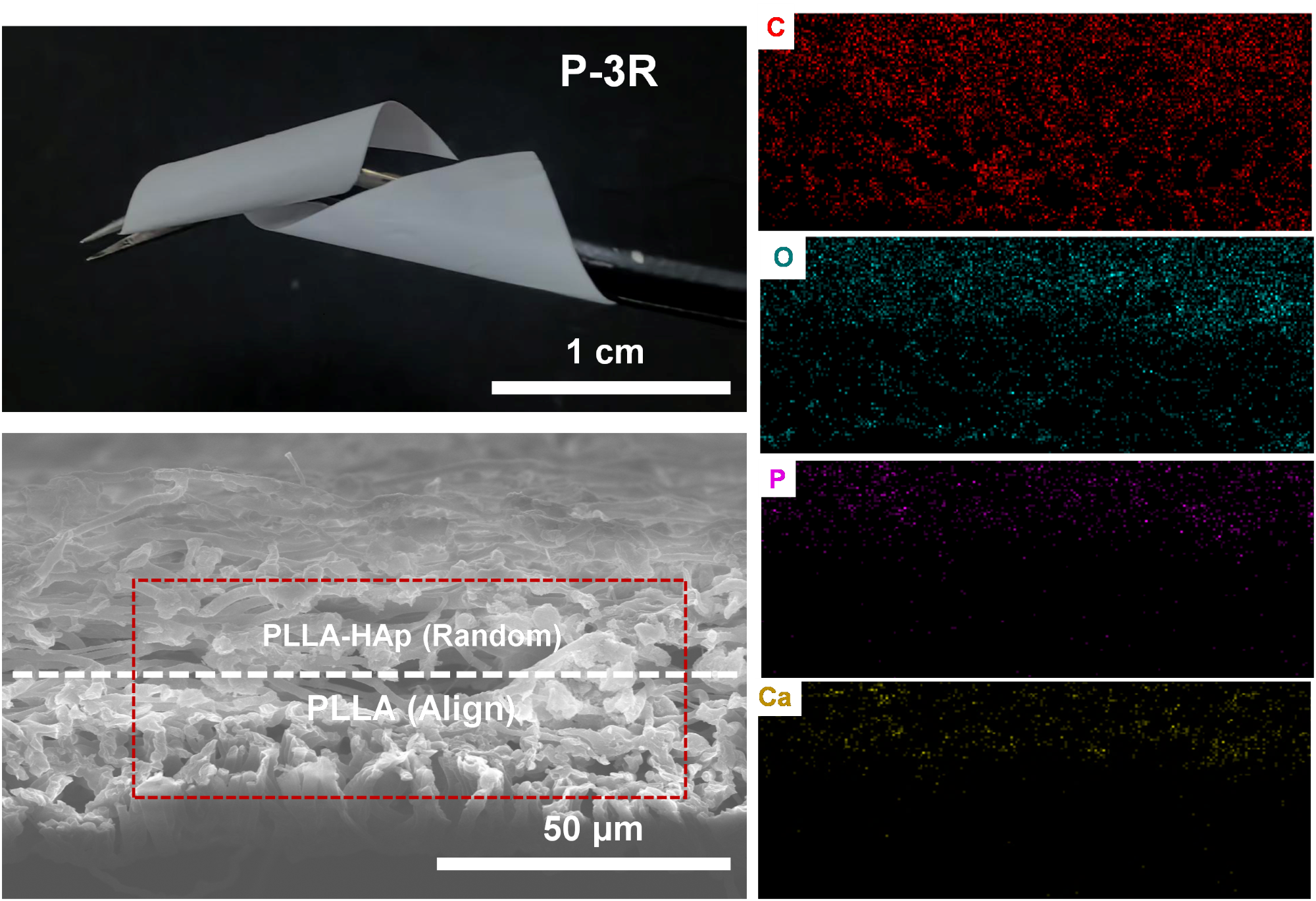


**Figure S13**. Digital images and cross-sectional morphology of P-3R fiber membranes, along with corresponding elemental mapping.


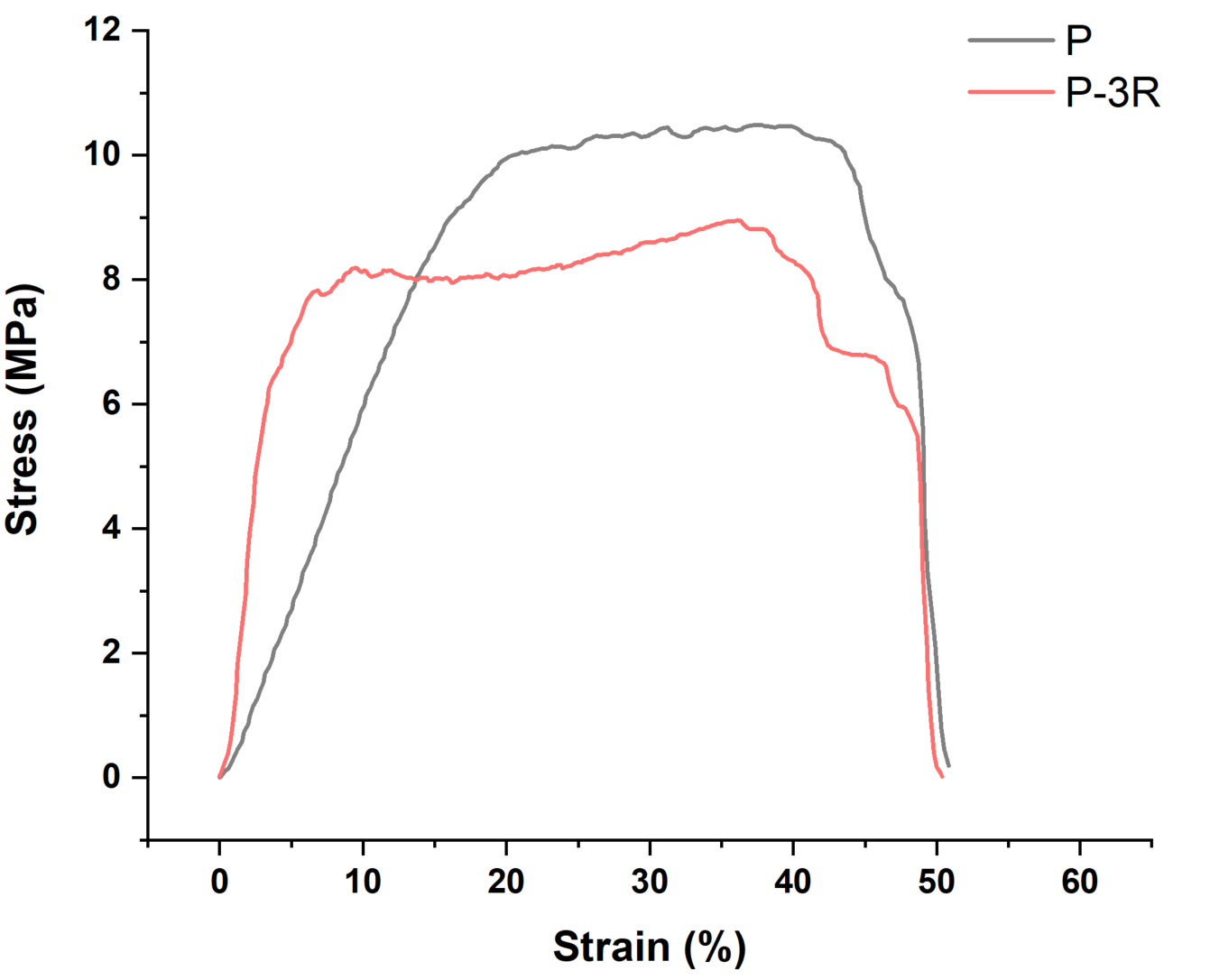


**Figure S14**. Mechanical properties of P and P-3R fiber membranes.


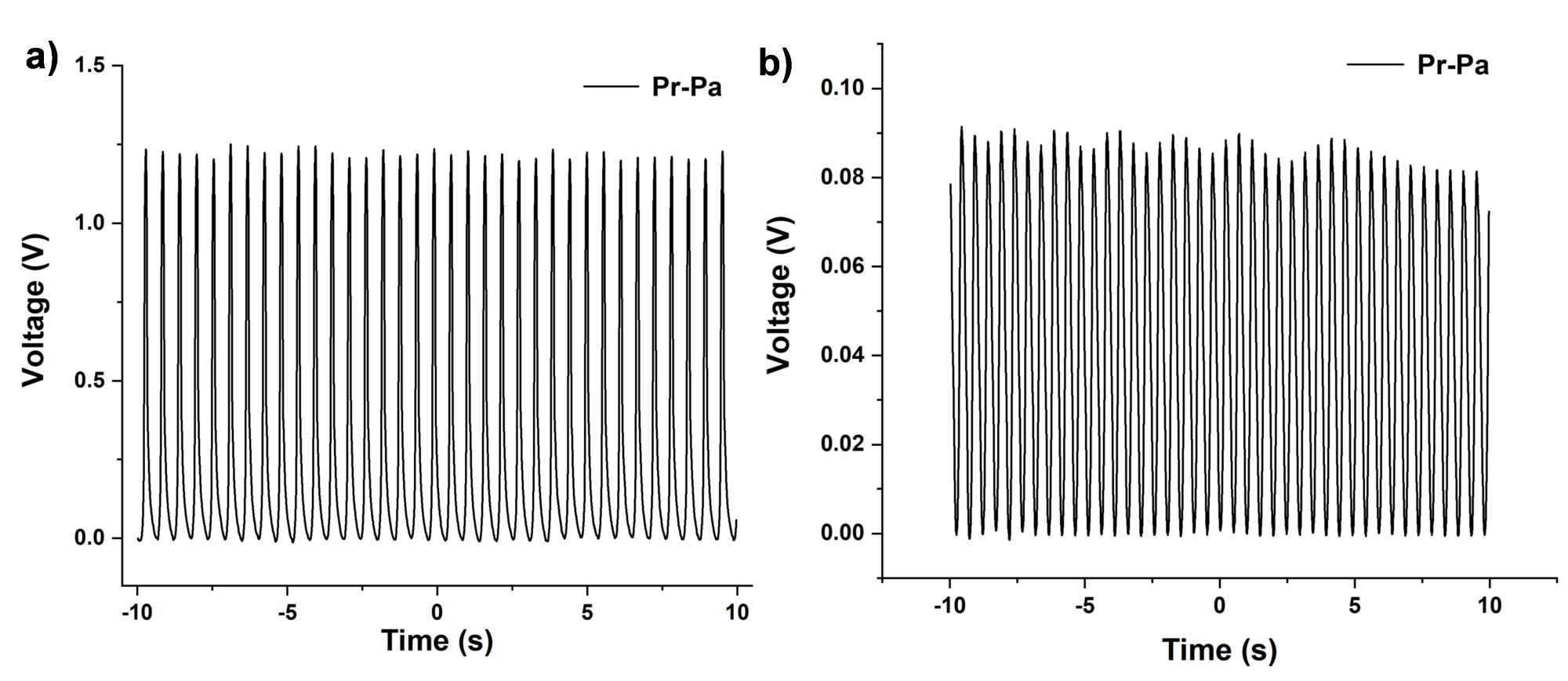


**Figure S15**. Piezoelectric output of Pr-Pa fiber membranes with topological structures under a) impact and b) bending modes.


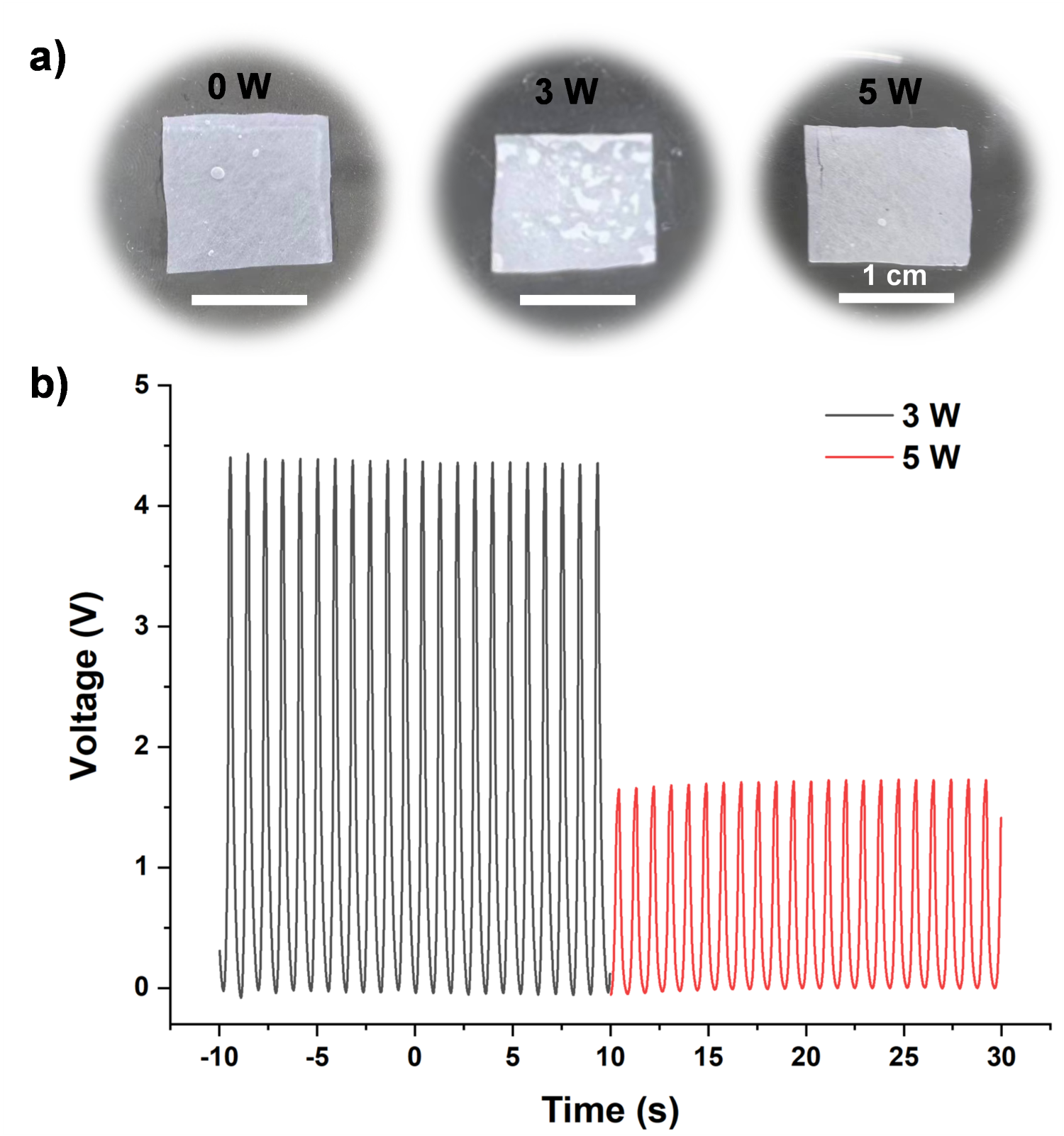


**Figure S16**. Degradation of P-3R fibers over different time periods (37 °C, PBS) and piezoelectric output during the degradation process.


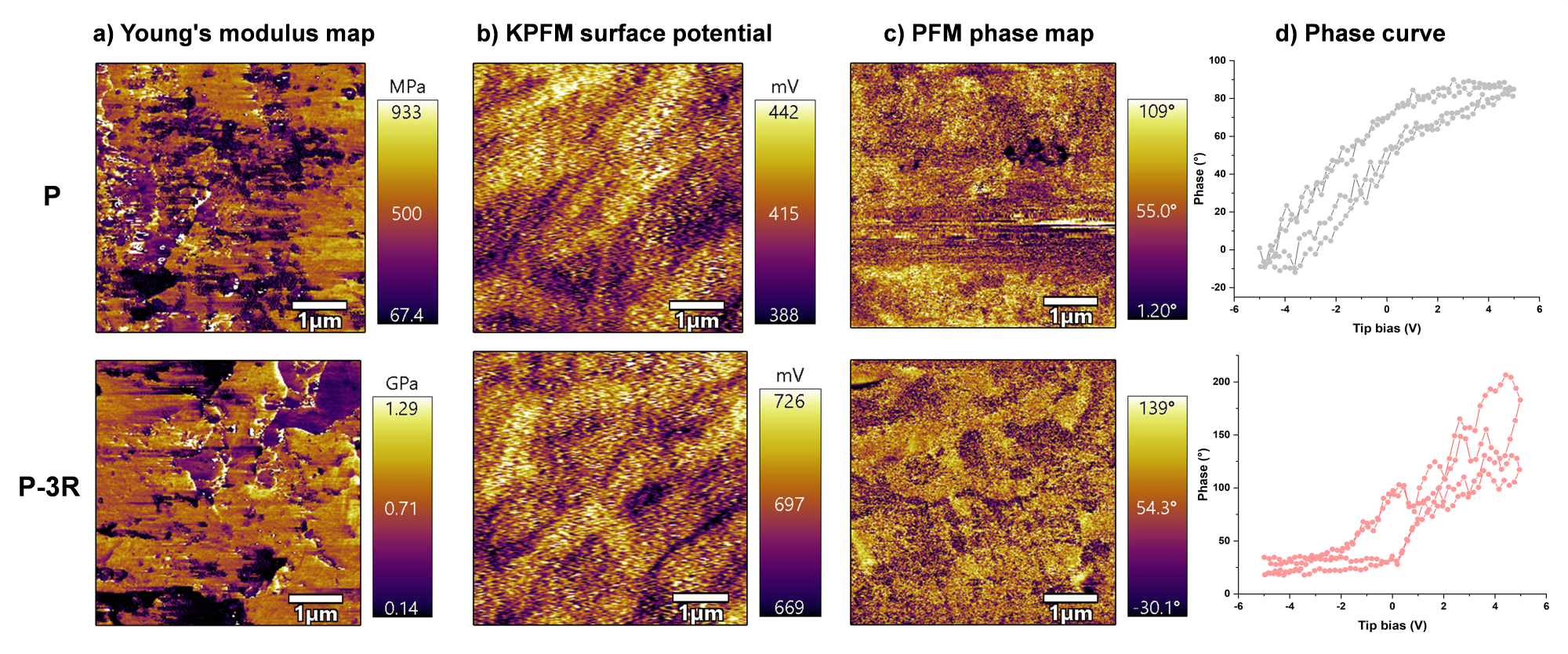


**Figure S17**. a) Young’s modulus maps, b) surface potential, c) PFM phase maps, and d) phase curves for P and P-3R fibers.


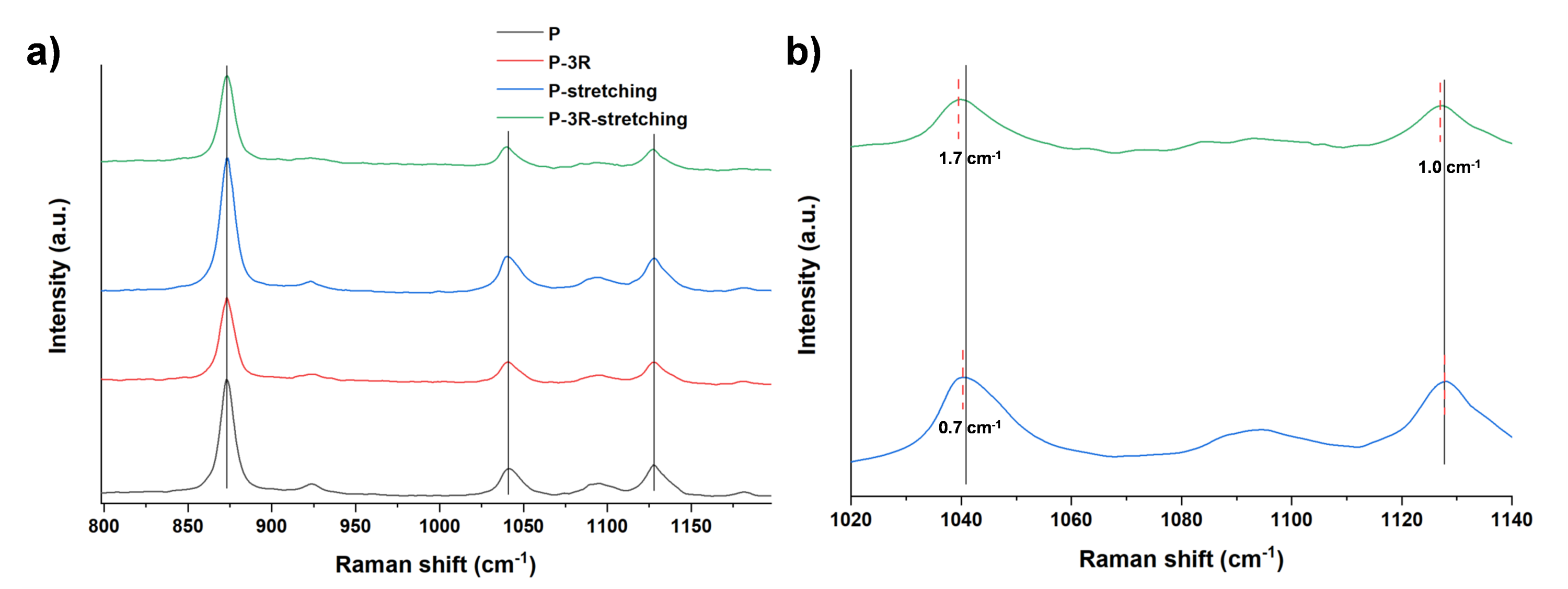


**Figure S18**. a) Raman spectra and b) magnified views of P and P-3R fiber membranes before and after stretching.


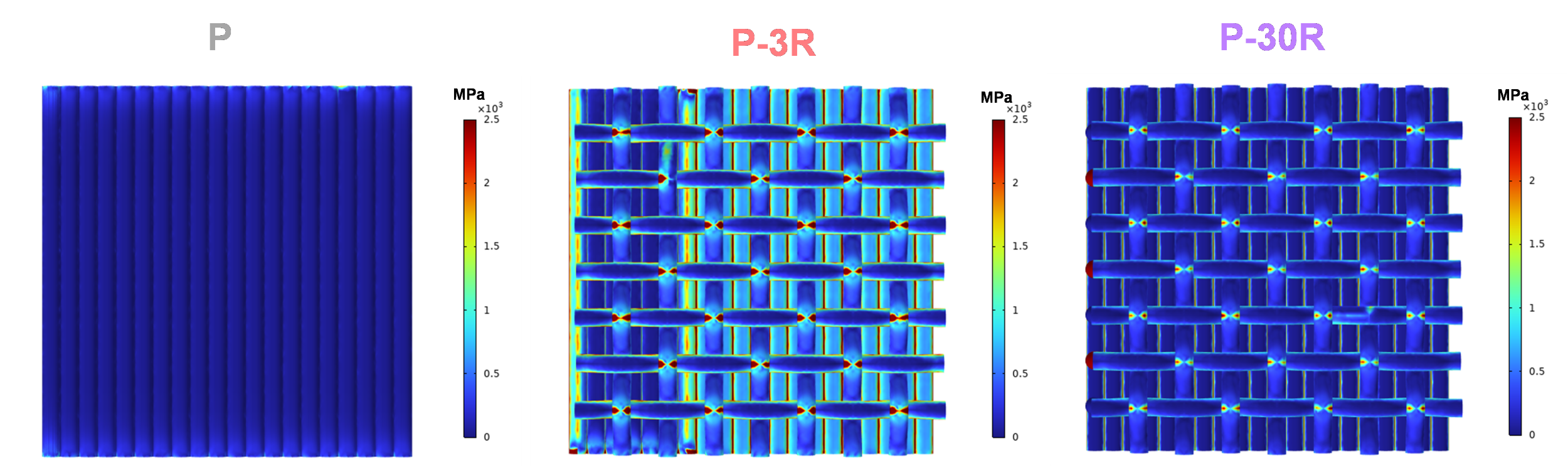


**Figure S19**. Top view of stress distribution for P, P-3R, and P-30R scaffolds in COMSOL analysis.


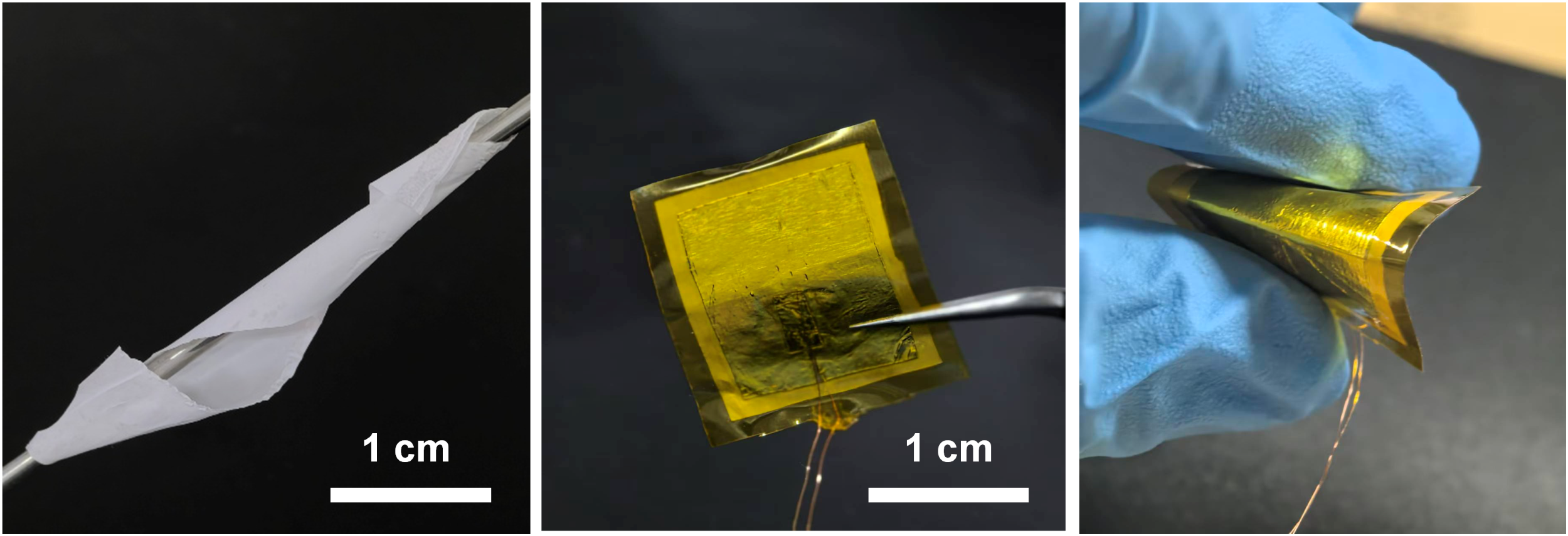


**Figure S20**. Digital photographs of the P-3R fiber membrane and the P-3R fiber device.





**Figure S21**. Simultaneous recording of ECG and piezoelectric signals over a 100 s period.





**Figure S22**. Comparison of heart rate derived from ECG and piezoelectric signals across five consecutive 20 s time windows using a window-based counting method.





**Figure S23**. Piezoelectric signals before and after applying a 0.5 Hz high-pass filter.

**Table S1**. Overview of the composition, structural characteristics, and piezoelectric properties of recent state-of-the-art biomaterials.

| Materials | Key  structure | Piezoelectric coefficients (pC/N) | Voltage density  (V/cm^2^)^*^ | References |
| --- | --- | --- | --- | --- |
| **PLLA/HAp** | **Topological fibrous scaffold** | **5.0 (d_33_​)** | **4.17** | **This work** |
| PLLA | Aligned fibers | 7.2 (d_33_​) | 3.78 | Ref. 40 |
| PLLA/BaTiO_3_ | Oriented fibers | 21.3 (d_33_​) | 1.02 | Ref. 14 |
| PLLA/Glycine | Core/shell nanofibers | 2.21 (d_33_) | 0.60 | Ref. 15 |
| PLLA/ZnO | Oriented fibers | 3.0 (d_33_) | 2.52 | Ref. 16 |
| PVDF/DA | Core-shell nanofibers | 59.4 (d_33_​) | 8.10 | Ref. S1 |
| PDA@BaTiO_3_/PVDF | Core-shell nanofibers | 24.1 (d_33_​) | 5.69 | Ref. S2 |
| PVDF/MXene | Confined orientation | 61.7 (d_33_​) | 1.09 | Ref. S3 |
| Tripeptides | Self-assembly crystals | 24.0 (d_33_​) | 2.27 | Ref. S4 |
| Silk Fibroin | Size-optimized nanofibers | 18.0 (d_33_​) | 1.11 | Ref. S5 |

Note: *Values were calculated and normalized based on the reported open-circuit voltages and the active areas of the devices for fair comparison.

**References**

[S1] T. Li, M. Qu, C. Carlos, L. Gu, F. Jin, T. Yuan, X. Wu, J. Xiao, T. Wang, W. Dong, X. Wang, Z.-Q. Feng, *Adv. Mater.* **2021**, 33, 2006093.

[S2] Y. Su, C. Chen, H. Pan, Y. Yang, G. Chen, X. Zhao, W. Li, Q. Gong, G. Xie, Y. Zhou, S. Zhang, H. Tai, Y. Jiang, J. Chen, *Adv. Funct. Mater.* **2021**, 31, 2010164.

[S3] L. Jin, Y. Ao, T. Xu, J. Zhang, Y. Zou, B. Lan, S. Wang, W. Deng, W. Yang, *J. Mater. Chem. A* **2025**, 13, 14446.

[S4] X. Yang, S. Liu, H. Lu, Y. Wang, H. Zhang, W. Ji, H. Wang, *Adv. Mater.* **2025**, 37, 2417409.

[S5] L. Jin, Y. Tai, J. Nam, *Nano Energy* **2024**, 132, 110367.
